# Exploring novel microbial metabolites and drugs for inhibiting *Clostridioides difficile*

**DOI:** 10.1128/msphere.00273-24

**Published:** 2024-06-28

**Authors:** Ahmed A. Abouelkhair, Mohamed N. Seleem

**Affiliations:** 1 Department of Biomedical Sciences and Pathobiology, Virginia-Maryland College of Veterinary Medicine, Virginia Polytechnic Institute and State University, Blacksburg, Virginia, USA; 2 Center for One Health Research, Virginia Polytechnic Institute and State University, Blacksburg, Virginia, USA; 3 Department of Bacteriology, Mycology, and Immunology, Faculty of Veterinary Medicine, University of Sadat City, Sadat City, Menoufia, Egypt; NC State University, Raleigh, North Carolina, USA

**Keywords:** screening, microbiota, MIC, microbial metabolites, *Clostridioides difficile*

## Abstract

*Clostridioides difficile* is an enteric pathogen that can cause a range of illnesses from mild diarrhea to pseudomembranous colitis and even death. This pathogen often takes advantage of microbial dysbiosis provoked by antibiotic use. With the increasing incidence and severity of infections, coupled with high recurrence rates, there is an urgent need to identify innovative therapies that can preserve the healthy state of the gut microbiota. In this study, we screened a microbial metabolite library against *C. difficile*. From a collection of 527 metabolites, we identified 18 compounds with no previously identified antimicrobial activity and metabolites that exhibited potent activity against *C. difficile* growth. Of these 18 hits, five drugs and three metabolites displayed the most potent anti-*C*. *difficile* activity and were subsequently assessed against 20 clinical isolates of *C. difficile*. These potent agents included ecteinascidin 770 (minimum inhibitory concentration against 50% of isolates [MIC_50_] ≤0.06 µg/mL); 8-hydroxyquinoline derivatives, such as broxyquinoline and choloroquinaldol (MIC_50_ = 0.125 µg/mL); ionomycin calcium salt, carbadox, and robenidine hydrochloride (MIC_50_ = 1 µg/mL); and dronedarone and milbemycin oxime (MIC_50_ = 4 µg/mL). Unlike vancomycin and fidaxomicin, which are the standard-of-care anti-*C*. *difficile* antibiotics, most of these metabolites showed robust bactericidal activity within 2–8 h with minimal impact on the growth of representative members of the normal gut microbiota. These results suggest that the drugs and microbial metabolite scaffolds may offer alternative avenues to address unmet needs in *C. difficile* disease prevention and treatment.

**IMPORTANCE:**

The most frequent infection associated with hospital settings is *Clostridioides difficile*, which can cause fatal diarrhea and severe colitis, toxic megacolon, sepsis, and leaky gut. Those who have taken antibiotics for other illnesses that affect the gut's healthy microbiota are more susceptible to *C. difficile* infection (CDI). Recently, some reports showed higher recurrence rates and resistance to anti-*C. difficile*, which may compromise the efficacy of CDI treatment. Our study is significant because it is anticipated to discover novel microbial metabolites and drugs with microbial origins that are safe for the intestinal flora, effective against *C. difficile*, and reduce the risk of recurrence associated with CDI.

## INTRODUCTION

Worldwide, hospitals and healthcare facilities frequently experience antibiotic-associated diarrhea due to *Clostridioides difficile* (1, 2). In the United States, *C. difficile* caused over 12,800 deaths and approximately 223,900 hospital admissions in 2017 alone. In addition, *C. difficile* accounted for 33,200 deaths globally in 2019, which imposed a substantial burden on the healthcare sector (3). *C. difficile* continues to be an urgent threat to the healthcare system and causes a substantial financial burden that accounts for $4.8 billion annually (4). Consequently, the bacterium has been categorized by the U.S. Centers for Disease Control and Prevention (CDC) as an urgent threat (5, 6).

Due to its role in *C. difficile*’s colonic proliferation, intake of antibiotics is a significant risk factor for *C. difficile* infection (CDI)(7). Because it provides resistance to *C. difficile* colonization, the healthy gut microbiota serves as a protective barrier against CDI (8). When broad-spectrum antibiotics are used and the usual makeup of the gut microbiota is disturbed, patients are more susceptible to intestinal colonization by *C. difficile* (9, 10). This leads to an increase of deadly toxins being produced by *C. difficile* during spore germination and vegetative cell outgrowth (11). The severe gastrointestinal inflammation that *C. difficile* toxins cause provides the ideal conditions for the bacteria to continue to survive and the infection to persist (12, 13).

Moreover, the main factor responsible for CDI recurrence and spread, spores, has a strong correlation with the clinical severity of CDI (14). The germination and vegetative development of *C. difficile* spores can be inhibited by a robust and varied microbiome. Spore germination and toxin production will transpire if the metabolic and microbiological environment of the gut has been disturbed (15, 16). Individuals who are initially diagnosed with CDI have a 20% probability of experiencing a recurrence, and between 19% and 28% of them do not respond well to therapy after taking antibiotics (17, 18).

The FDA-approved anti-CDI armamentarium includes three antibiotics, metronidazole, vancomycin, and fidaxomicin. Metronidazole should only be used for scenarios that are not severe when vancomycin and fidaxomicin are not readily accessible (19). Treatment failure rates are higher with vancomycin and metronidazole, frequent CDI recurrence, disruption of the normal intestinal microbiome, and promotion of vancomycin-resistant enterococci (VRE) overgrowth (20–23). Fidaxomicin is the only antibiotic approved against *C. difficile* in the last three decades (24). Despite its selectivity toward *C. difficile* over the gut microbiota, fidaxomicin is associated with multiple cases of treatment failure (25–27). In addition, fecal microbiota transplant (FMT) has emerged as a non-antibiotic therapy for CDI to restore the normal microbiome population. Currently, two live biotherapeutic products (LBPs) are approved by the FDA: Ferring’s Rebyota and Seres' Vowst (28, 29). However, FMT lacks standardization of the treatment protocol and poses a serious risk of transmitting infectious agents that could be fatal especially in immunocompromised and elderly patients (30–33). Therefore, there is an unmet need to identify new effective therapeutics with high selectivity against *C. difficile* rather than the beneficial microbiota.

Despite recent technological advances, discovery of a novel drug can take up to 15 years and cost an average of $2–3 billion (34). We screened a library of microbial metabolites and drugs to identify novel anti*-C*. *difficile* metabolites of microbial origin and novel drugs that would restrict the recurrence rate and maintain healthy gut microbiota. Microorganisms are traditionally considered abundant reservoirs of rare bioactive compounds. Microbial metabolites have demonstrated efficacy as antibacterial, anti-tumor, enzyme inhibition, and anti-inflammatory agents (35, 36). In this study, eight promising microbial metabolites and drugs against *C. difficile* were identified. The antibacterial activity of these metabolites against a panel of clinically relevant *C. difficile* strains was evaluated. Additionally, the activity of these metabolites against representative members of the human gut microbiota was investigated.

## MATERIALS AND METHODS

### Bacterial strains and reagents

Isolates of *C. difficile* and gut normal microbiota were acquired from the CDC (Atlanta, GA, USA), the American Type Culture Collection (ATCC), and the Biodefense and Emerging Infections Research Resources Repository (BEI Resources) in Manassas, VA, USA. (Table S1 and S2). Phosphate-buffered saline (PBS) (Corning, NY, USA), yeast extract (Fisher Scientific, Hampton, NH, USA), tryptic Soy Broth (TSB) de Man, Rogosa and Sharpe (MRS) broth, and brain heart infusion (BHI) broth were purchased commercially (Becton, Dickinson, and Company, NJ, USA). L-cysteine (Alfa Aesar, Haverhill, MA, USA), vitamin K, and hemin (Sigma-Aldrich, Saint Louis, MO, USA) were purchased commercially.

### Compounds and library

The microbial metabolite library, which contains a unique collection of 527 microbial metabolites and drugs (HY-L084), was purchased from MedChemExpress (Princeton, NJ, USA).

Ionomycin (calcium salt) and carbadox (Cayman Chemicals, Ann Arbor, MI, USA); broxyquinoline (Alfa Aesar, Haverhill, MA, USA); dronedarone (Fisher Scientific, Hampton, NH, USA); robenidine hydrochloride and ecteinascidin 770 (ET-770) (MedChemExpress, Princeton, NJ, USA); milbemycin oxime (Targetmol, Wellesley Hills, MA, USA); and choloroquinaldol (Ambeed, Arlington Heights, IL, USA) were purchased commercially. Fidaxomicin (Biosynth Carbosynth, Louisville, KY, USA) and vancomycin hydrochloride (Gold Biotechnology, Olivette, MO, USA) were included as control antibiotics.

### Library screening against *C. difficile*

A single screening of the microbial metabolite library was conducted against *C. difficile* ATCC BAA-1870 at a fixed concentration of 16  µM, using the broth microdilution method as reported previously (37–40). Briefly, the bacterial strain was cultured onto BHI agar supplemented with yeast extract, vitamin K1, hemin, and L-cysteine (BHIS) and incubated anaerobically at 37°C for 48 h. Test agents were added to 0.5 McFarland bacterial solution, diluted in BHIS broth (~5  ×  10^5^ CFU/mL), and added to 96well plates. The plates were kept in an anaerobic environment at 37°C for 48 h. The OD_600_ was measured using a SpectraMax i3 Multi-Mode Microplate Reader (Molecular Devices, Sunnyvale, CA, USA). With regard to the dimethyl sulfoxide (DMSO) negative control, the percentage of inhibition was calculated for each tested agent. "Hits" were metabolites with an inhibition level of 90% and verified to be active and purchased commercially. The metabolites that showed no action had been eliminated from the study. GraphPad Prism version 8.0 was used to illustrate the growth inhibition percentage.

### Activity of selected hits against *C. difficile*

The hits were subsequently tested against *C. difficile* ATCC BAA-1870 to ascertain the accurate minimum inhibitory concentrations (MICs) of the active hits from the library screening, using the broth microdilution method as previously described (40–46). Briefly, serial dilutions of the active hits and the control antibiotics (vancomycin and fidaxomicin) were anaerobically incubated with bacterial suspensions (~5  ×  10^5^ CFU/mL) at 37  °C for 48 h. The 96-well microtiter plates were then examined for growth, and the MIC was determined as the lowest concentration that completely inhibited the bacterial growth as determined by visual inspection of plates.

Based on the MIC data of the active hits (MIC range ≤4 µg/mL), eight microbial metabolites and drugs (ET770, two 8-hydroxyquinoline derivatives [broxyquinoline and choloroquinaldol], ionomycin calcium salt, carbadox, robenidine hydrochloride, dronedarone, and milbemycin oxime) were further investigated. These eight molecules were purchased and tested against a panel of 20 *C*. *difficile* clinical isolates, as described above. The MIC_50_ and MIC_90_ values (the concentration of test agent that inhibited 50% and 90% of the strains respectively) were determined. The experiment was done in triplicates.

### Time-kill assay against *C. difficile*

A time-kill assay was performed against *C. difficile* ATCC BAA-1870 to evaluate the killing kinetics of the hits. The assay was performed as previously described (37, 46, 47). Briefly, an overnight culture of *C. difficile* in the logarithmic growth phase was diluted in pre-reduced BHIS ~ 10^6^ CFU/mL (1:100) and exposed to 5 × MIC of the positive hits and the control antibiotics vancomycin or fidaxomicin. Aliquots (10 µL) were taken from each treatment at specific time points, diluted, and plated onto pre-reduced BHIS agar plates to determine the viable bacterial count for up to 24 h. The experiment was done in triplicates.

### *In vitro* activity against representative members of the human gut microbiota

The eight active metabolites and drugs were assessed for their antibacterial activity against reference strains of the human normal gut flora, following established procedures (43, 46, 48). A bacterial solution equivalent to a 0.5 McFarland standard was prepared and diluted in MRS broth (for *Lactobacillus* strains) and BHIS broth (for *Bacteroides* and *Bifidobacterium* species) or TSB (for strains of *Enterococcus*) to achieve a bacterial concentration of ~5 × 10^5^ CFU/mL. Serial dilutions of test agents were incubated with bacteria before determining the MICs. Anaerobic incubation was used for *Bacteroides* and *Bifidobacterium* species, whereas 5% CO_2_ was used for *Lactobacillus*. Enterococci were incubated aerobically. The experiment was done in triplicates.

### Cytotoxicity assessment against vero cells

To evaluate the potential toxic effect of the metabolites, cytotoxicity was assessed against Vero cells as has been described previously (37, 38). Briefly, Vero cells were exposed to metabolites and incubated for 24 h. DMSO was included as a negative control. MTS/PMS reagent was subsequently added to the cells and incubated for 2 h. The absorbance values at OD_490_ were measured using a Biotek Synergy H1 Hybrid microplate reader. The data were presented as percent viable cells (mean ± standard deviation) relative to DMSO-treated cells.

## RESULTS AND DISCUSSION

First-line antimicrobials for *C. difficile* failed to guarantee clinical cure; patients frequently experience recurrent CDI (49–51). Therefore, researchers have been exploring alternative treatments for these challenging infections. Today, a large variety of bioactive metabolites are utilized in medicine, and many antibiotics with microbial origins are available on the market (52). In light of this strategy, and the detrimental effects that antibiotics have on the gut microbiome, we focused on using compounds generated by microorganisms to target *C. difficile* in an effort to find novel scaffolds with anti-*C*. *difficile* action.

### Screening a library of microbial metabolites against *C. difficile*

**Fig 1:**
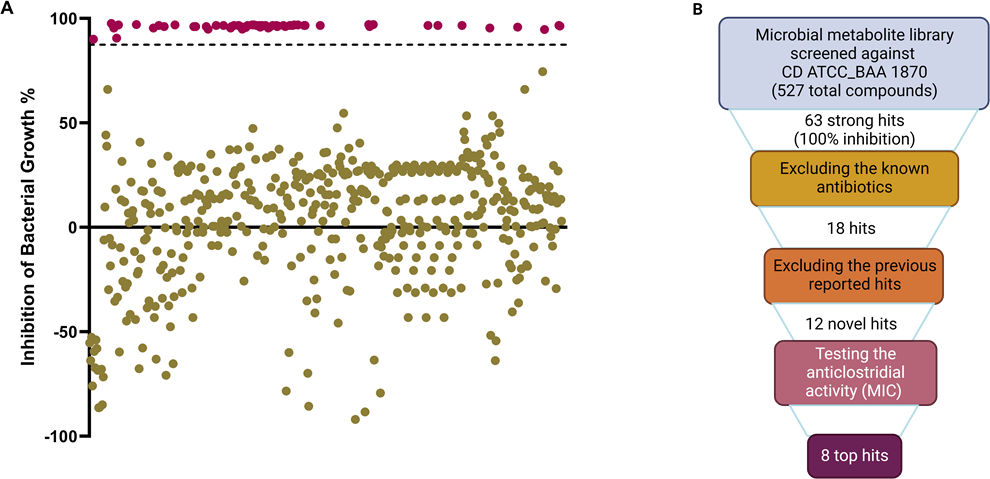
**(A**) Screening the library of microbial metabolites against *C. difficile* identifies novel lead scaffolds. Results from screening 527 microbial metabolites at a concentration of 16 µM against *C. difficile* ATCC BAA-1870. Agents designated as hits (red in color) showed >90% reduction of bacterial growth. (**B**) A flow chart showing how many compounds are evaluated at each step.

The microbial metabolite library (containing 527 compounds) was screened against *C. difficile* ATCC BAA-1870 at a concentration of 16 µM using the broth microdilution assay. According to the CLSI, broth microdilution is not a recommended method for antimicrobial susceptibility testing (AST) of *C. difficile*, but agar dilution is. However, broth microdilution is more accurate and practical especially in case of screening of drug libraries. The advantages of broth microdilution include reproducibility, the small amount of sample required, and the low cost allowing large numbers of replicates (53). In addition, AST via broth microdilution for *C. difficile* is well established and reported in multiple publications (46, 54–56). The initial screening identified 63 compounds that inhibited the growth of *C. difficile* (>90% of bacterial growth was inhibited) at 16 µM (Fig. 1; Table S3). After excluding antibacterial agents, we selected 18 metabolites (Table 1).

**TABLE 1:** Active hits identified from initial screening against *C. difficile* ATCC BAA-1870

No.	Compound name	Compound structure	Source	Class
**1**	ET-770	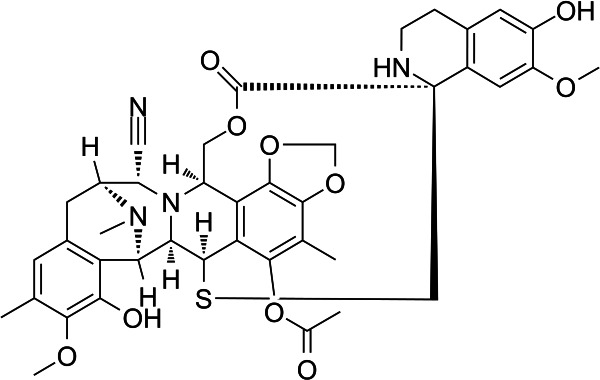	*Ecteinascidia thurstoni*	Anticancer
**2**	Chlorquinaldol	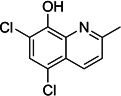		Topical antiseptic and for vaginal infections
**3**	Broxyquinoline	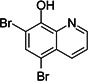		Antiprotozoal
**4**	Ionomycin (calcium salt)	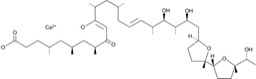	*Streptomyces conglobatus*	Calcium ionophore
**5**	Carbadox	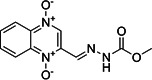		Feed additive (Growth promoter)
**6**	Robenidine hydrochloride	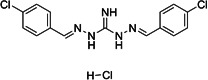		Coccidiostat drug
**7**	Dronedarone	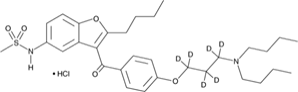		Antiarrhythmic
**8**	Milbemycin oxime	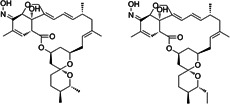	*Streptomyces hygroscopicus*	Anthelmintic
**9**	Ivermectin	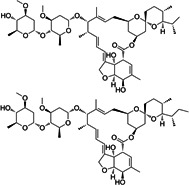	*Streptomyces avermitilis*	Anthelmintic
**10**	Ginsenoside C-K	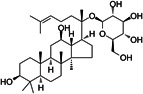	Soil bacteria	Anti-inflammatory and antineoplastic
**11**	Avermectin B1	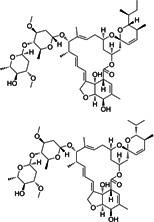	*Streptomyces avermitilis*	Anthelmintic
**12**	GW 501516	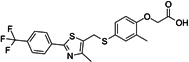		Metabolic modulator
**13**	Tinidazole	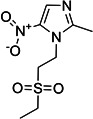		Antiprotozoal
**14**	Tioconazole	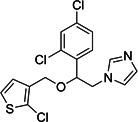		Antifungal
**15**	Miconazole (nitrate)	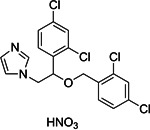		Antifungal
**16**	Chloroxine	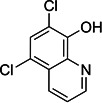		Chelating agent, anti-seborrheic
**17**	Lithocholic acid	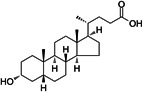		Secondary bile acid
**18**	Octenidine (dihydrochloride)	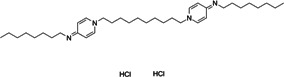		Antiseptic

Six metabolites were excluded because they were previously reported, including tinidazole, tioconazole, miconazole, and choloroxine (48); lithocholic acid (57); and octenidine dihydrochloride (58). The other 12 unique compounds, which were not reported against *C. difficile* previously, were selected for further investigation (Table 2). Afterwards, the 12 metabolites were screened against *C. difficile,* and their MICs were determined. Of the 12 hits, eight metabolites and drugs (ET-770, broxyquinoline, chlorquinaldol, ionomycin, carbadox, robenidine hydrochloride, dronedarone, and milbemycin oxime) displayed the most potent activity against *C. difficile* ATCC BAA-1870 with MIC values ranging from ≤0.06 μM to 4 µM. Due to their unique nature and lack of prior research, these promising hits were chosen for further investigation.

**TABLE 2:** Minimum inhibitory concentrations (MICs [μM]) of the microbial metabolites against *C. difficile* ATCC BAA-1870

No.	Metabolite ID	MIC (μM)	MIC (μg/mL)
1	ET-770	≤ 0.25	≤ 0.19
2	Chlorquinaldol	2	0.91
3	Broxyquinoline	4	1.21
4	Ionomycin (calcium salt)	4	2.99
5	Carbadox	4	1.05
6	Robenidine hydrochloride	8	2.97
7	Dronedarone	8	4.45
8	Milbemycin oxime	4	4.39
9	Ivermectin	16	14
10	Ginsenoside C-K	16	9.97
11	Avermectin B1	16	27.71
12	GW 501516	16	14.51
13	Vancomycin		1
14	Fidaxomicin		≤0.0078

### *In vitro* activity of the active hits against a panel of *C. difficile* strains

Next, we evaluated the anti-*C*. *difficile* activity of eight active hits against 20 *C*. *difficile* clinical isolates (Table 3). ET-770 showed strong anti-*C*. *difficile* activity (MIC_50_ = 0.06 µg/mL) and inhibited the growth of the tested isolates at doses ranging from 0.008 to 0.5 μg/mL. ET-770 is naturally produced from *Ecteinascidia thurstoni*, which has potent anticancer qualities and can make human lung cancer cells more sensitive to anoikis (59). ET-770 possesses antibacterial activity against *Bacillus subtilis*, methicillin-resistant *S. aureus* (MRSA), and *Escherichia coli*, with MIC values of 1.01, 2.02, and 32.43 µM, respectively (60). Furthermore, with (half-maximal inhibitory concentration [IC_50_] of 0.13 µM), ET-770 previously demonstrated extremely strong activity against *Mycobacterium tuberculosis* (60).

**TABLE 3:** The MIC values selected for microbial metabolite hits and control anti-*C*. *difficile* agents against clinical isolates of *C. difficile^a^*

*C.difficile* strain ID	Microbial metabolite hits, minimum inhibitory concentration (MIC) (μg /mL)								Control antibiotics	
	ET-770	CHO	BRO	ION	CAR	ROB	DRO	MBO	VAN	FDX
CD-1 (Ribotype-027)	0.125	0.125	0.25	1	2	1	4	4	2	0.06
CD-6 (Ribotype-027)	0.125	0.125	0.25	1	0.5	1	4	4	2	0.06
CD-10(Ribotype-027)	0.125	0.125	0.125	1	2	1	4	4	2	0.03
CD-12	0.125	0.25	0.125	2	2	1	4	4	1	0.03
CD-13	0.06	0.25	0.03	1	1	2	4	8	0.5	≤0.0078
CD-15	0.06	0.125	0.25	1	1	2	4	4	0.5	0.015
CD-22	0.125	0.125	0.125	1	2	2	2	8	0.5	≤0.0078
CD-23	0.06	0.125	0.125	2	2	1	8	4	0.25	0.06
CD-24	0.125	0.06	0.015	0.5	1	1	2	4	0.25	≤0.0078
CD-26 (Ribotype-027)	0.125	0.125	0.125	1	2	1	4	4	1	0.06
CD-28	0.03	0.125	0.06	1	2	1	8	8	0.5	0.015
CD-29 (Ribotype-027)	0.06	0.06	0.06	2	0.5	1	2	4	1	≤0.0078
CD-30	0.125	0.5	0.25	2	2	2	8	4	0.5	0.015
NR-49302	0.06	0.125	0.06	2	2	1	4	4	0.5	0.03
NR-49304	0.06	0.06	0.03	4	1	1	2	8	0.5	≤0.0078
NR-49308	0.125	0.06	0.06	2	1	1	4	4	0.5	≤0.0078
NR-49319	0.06	0.06	0.25	2	0.5	1	8	8	0.5	0.03
ATCC BAA-1870 (Ribotype-027)	0.125	0.125	0.125	1	2	2	4	8	1	≤0.0078
ATCC 630	0.03	0.25	1	0.5	1	1	4	4	0.25	≤0.0078
ATCC 43255	0.06	0.06	0.25	1	1	1	4	4	0.25	0.03
MIC_50_	**0.06**	**0.125**	**0.125**	**1**	**1**	**1**	**4**	**4**	**0.5**	**0.015^*b*^**
MIC_90_	**0.125**	**0.25**	**0.25**	**2**	**2**	**2**	**8**	**8**	**2**	**0.06**

^*a*^ ET-770 ecteinascidin 770; CHO, chlorquinaldol; BRO, broxyquinoline; ION, ionomycin; CAR, carbadox; ROB, robenidine hydrochloride; DRO, dronedarone; MBO, milbemycin oxime; VAN, vancomycin; FDX, fidaxomicin.

^*b*^ The minimum inhibitory concentration (MIC50) against 50% of isolates is represented by the bold values.

The derivatives of 8-hydroxyquinoline, broxyquinoline, and chlorquinaldol, exhibited strong anti-*C*. *difficile* activity with an MIC_50_ value of 0.125 µg/mL, which was comparable to the MIC of fidaxomicin (Table 3). Chlorquinaldol is a common chelating agent that possesses robust antibiofilm action against *Pseudomonas aeruginosa* and *S. aureus* biofilms, with MIC values ranging from 0.016 to 0.5 μg/mL against staphylococci (61, 62). Chlorquinaldol is administered topically as a cream for skin infections (63). Broxyquinoline is an antiprotozoal agent that is poorly absorbed from the gastrointestinal tract (64, 65); broxyquinoline exhibited potent anti-*C*. *difficile* activity against the tested isolates (MIC_50_ = 0.125 µg/mL). Therefore, broxyquinoline’s potency and low absorption make it an intriguing molecule to further pursue for development as a novel anti-*C*. *difficile* agent. Against *Staphylococcus epidermidis*, *S. aureus*, and *Acinetobacter baumannii*, broxyquinoline exhibited modest antibacterial activity with MIC values of 12.5 µM (4 µg/mL) (66).

Ionomycin is a calcium ionophore agent that was isolated from the bacterium *Streptomyces conglobatus*. Ionomycin exhibits high potency and selectivity against Gram-positive bacteria (67). Ionomycin can activate Ca^2+^/calmodulin-dependent kinase and phosphatase to induce gene expression (68). Ionomycin inhibited the tested *C. difficile* isolates at concentrations ranging from 0.5 to 4 μg/mL (MIC_50_ = 1 µg/mL), which was similar to the MIC values for vancomycin, the drug of choice for the treatment of CDI (Table 3).

It is interesting to note that the MIC values of ionomycin (MIC_50_ = 1 µg/mL and MIC_90_ = 2 µg/mL) were similar to those of vancomycin (MIC_50_ = 0.5 µg/mL and MIC_90_ = 2 µg/mL) against the tested strains of *C. difficile*, respectively. Similarly, broxyquinoline and chlorquinaldol (MIC_50_ = 0.125 µg/mL and MIC_90_ = 0.25 µg/mL) as well as ET-770 (MIC_50_ = 0.06 µg/mL and MIC_90_ = 0.125 µg/mL) exhibited MIC values that were comparable to fidaxomicin (MIC_50_ = 0.015 µg/mL and MIC_90_ = 0.06 µg/mL).

Carbadox is frequently used as a feed additive in swine to stimulate growth and manage swine dysentery. Additionally, carbadox inhibits the growth of spirochete bacteria isolated from dogs (69, 70). However, the drug has been associated with the development of tumors and birth defects in laboratory animals. Despite these concerns, carbadox displayed potent activity against the strains of *C. difficile* that were tested (MIC_50_ = 1 µg/mL) (Table 3).

Another promising discovery in our screening is robenidine hydrochloride, an anticoccidial drug with MIC values of 4.7 and 8.1 µM, respectively, that has been shown to be effective against vancomycin-resistant enterococci (VRE) and MRSA (71). Additionally, robenidine hydrochloride exhibits bactericidal activity against *A. baumannii* (MIC = 8 µg/mL) and *Acinetobacter calcoaceticus* (MIC = 2 µg/mL) (72). Robenidine is generally regarded as a safe drug with no observed genotoxicity or mutagenicity (73). Here, we report that robenidine hydrochloride exhibited strong antibacterial activity against *C. difficile* (MIC_50_ = 1 µg/mL), which was comparable to the control antibiotic vancomycin (Table 3).

Dronedarone is an antiarrhythmic drug that is used to treat cardiac arrhythmias (74). Dronedarone is a benzofuran molecule, the same as amiodarone; however, it lacks the iodine-containing groups that amiodarone is known to cause thyroid issues with. Because it has a methyl sulfonyl group in its structure, dronedarone is likewise more lipophilic and has a shorter half-life than amiodarone. Consequently, the probability of drug buildup is reduced in bodily tissues and organ toxicities, such as thyroid and lung concerns (75). Furthermore, dronedarone was previously found to possess antibacterial and antiparasitic activity against *S. aureus* and *Leishmania mexicana* (76, 77). Dronedarone also exhibits modest anti-*C*. *difficile* activity (MIC_50_ = 4 µg/mL) in these previous studies.

Finally, *Streptomyces hygroscopicus* produces milbemycin oxime, a macrocyclic lactone with broad-spectrum antiparasitic effect (78, 79). With MIC values of <8 µg/mL, milbemycin oxime has been found to have antimycobacterial activity (80). Comparably, milbemycin oxime showed some minor anti-*C*. *difficile* activity against the tested *C. difficile* isolates, with a MIC_50_ value of 4 µg/mL, which is four times greater than that of vancomycin (Table 3).

### Time-kill assay against *C. difficile*

**Fig 2:**
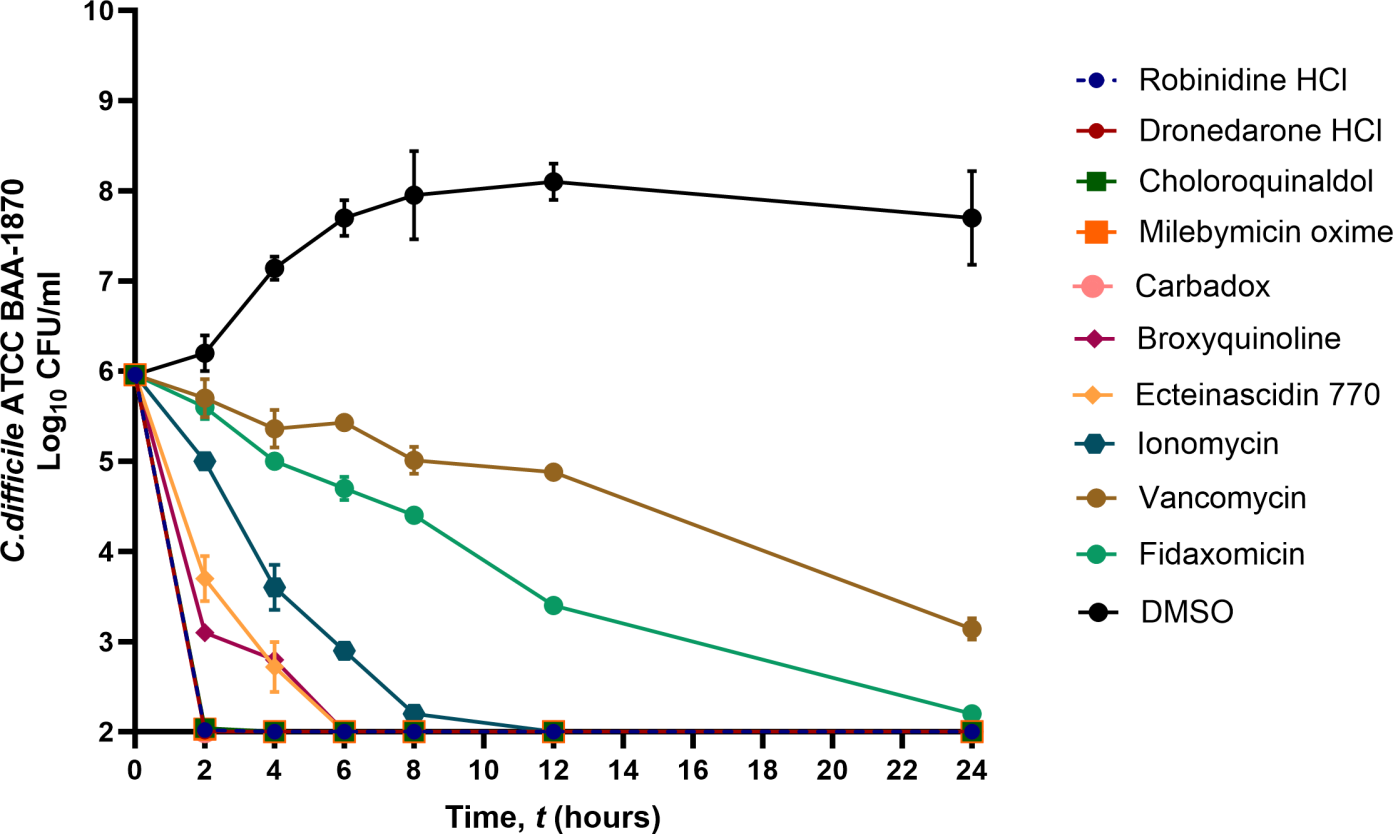
Time-kill assay of compounds from the library of microbial metabolites against *C. difficile*. The positive hits, vancomycin (VAN), and fidaxomicin’s (FDX) time-kill assays were performed at 5 × MIC against *C. difficile* ATCC BAA-1870. On pre-reduced BHIS medium, the start of *C. difficile* development was seen as CFUs during a 24-h period.

To determine the rate at which the positive hits from the library of microbial metabolites eliminated a high inoculum of *C. difficile*, we performed a time-kill assay. As shown in Fig. 2, in contrast to the control antibiotics, vancomycin and fidaxomicin, which eliminated *C. difficile* within 24 h, positive hits exhibited a bactericidal effect on *C. difficile* ATCC BAA-1870 at 5 × MIC within 2 h, with the exception of broxyquinoline and ET-770, which cleared *C. difficile* after 6 h. Ionomycin, by contrast, began to show a bactericidal effect after 8 h and completely cleared *C. difficile* after 12 h.

### *In vitro* activity against representative members of the human gut microbiota

Dysbiosis, induced by the administration of broad-spectrum antibiotics, can disrupt the normal intestinal microbial composition, which can lead to opportunistic infections, such as *C. difficile* colonizing the gut (81, 82). Therefore, it was crucial to ascertain if the active microbial metabolites detected by our screen may have a negative impact on commensal organisms that are often found in the normal gut microbiota. Targeted metabolites were tested for their antibacterial properties against species of *Bacteroides*, *Bifidobacterium*, *Lactobacillus*, and *Enterococcus*, which are typical of the human gut microbiota. The selected microbial metabolites and drugs exhibited poor activity against the representative members of normal microbiota strains tested. As presented in Table 4, ionomycin, dronedarone, broxyquinoline, and milbemycin oxime exhibited limited activity against *Bacteroides* with MIC values ranging from 8 to >256  µg/mL. By contrast, the remaining metabolites exhibited potent activity against species of *Bacteroides* (MIC values, ≤2 µg/mL). Regarding activity against *Bifidobacterium* species, ionomycin, broxyquinoline, and chlorquinaldol did not show significant inhibition (MIC values ranged from 64 to >256 µg/mL); dronedarone and milbemycin oxime were slightly active (MIC values ranged from 2 to 8 μg/mL), whereas the remaining metabolites significantly inhibited the growth of species of *Bifidobacterium* (MIC values, ≤2 µg/mL) similar to the control antibiotics vancomycin and fidaxomicin. Additionally, ionomycin, dronedarone, broxyquinoline, carbadox, milbemycin oxime, and chlorquinaldol exhibited limited activity against enterococcal species (MIC values ranged from 4 to >256µg/mL). ET-770 and robenidine hydrochloride exhibited more potent activity against enterococci compared with other compounds (MIC values, ≤2 µg/mL). With the exception of ET-770, all other microbial metabolites exhibited limited activity against species of *Lactobacillus*. In contrast, vancomycin and fidaxomicin significantly inhibited the growth of the majority of gut microbiota strains tested, which is in agreement with previous reports (16, 49, 50).

**TABLE 4:** Antibacterial activity (MIC in μg/mL) of hit compounds and control anti-*C*. *difficile* drugs against representative members of human normal gut microbiota

Bacterial strain ID	microbial metabolite hits, minimum inhibitory concentration (mic) (μg/ml)								Control antibiotics	
	ET-770	CHO	BRO	ION	CAR	ROB	DRO	MBO	VAN	FDX
*B. fragilis* HM- 709	≤2	≤2	8	>256	2	≤2	8	32	16	>256
*B. fragilis* HM- 718	≤2	≤2	16	>256	2	≤2	8	>256	32	>256
*B. dorei* HM- 719	≤2	≤2	8	>256	2	≤2	8	>256	16	>256
*Bif. breve* ATCC 15700	≤2	>256	>256	>256	16	≤2	8	4	≤2	≤2
*Bif. angulatum* HM- 1189	≤2	32	8	64	4	≤2	8	4	≤2	≤2
*Bif. adolescentis* HM- 633	≤2	32	64	16	4	≤2	8	4	≤2	≤2
*Bif. longum* HM- 845	≤2	128	>256	>256	4	≤2	8	8	≤2	≤2
*Bif. longum* HM- 846	≤2	32	128	64	8	≤2	8	8	≤2	≤2
*Bif. longum* HM- 847	≤2	64	32	128	16	≤2	8	8	≤2	≤2
*Bif. longum* HM- 848	≤2	64	64	256	4	≤2	8	8	≤2	≤2
*E. faecium* HM-202	≤2	>256	32	32	128	≤2	8	4	≤2	≤2
*E. fecalis* HM-970	≤2	128	16	16	128	≤2	4	8	>256	≤2
*L. casei* ATCC 334	≤2	>256	32	32	128	16	64	>256	>256	8
*L. brevis* ATCC-14864	≤2	>256	64	≥256	32	8	32	>256	>256	64
*L. rhamanosus* ATCC-53103	≤2	>256	>256	>256	>256	16	128	>256	>256	8

^*a*^ ET-770 ecteinascidin 770; CHO, chlorquinaldol; BRO, broxyquinoline; ION, ionomycin; CAR, carbadox; ROB, robenidine hydrochloride; DRO, dronedarone; MBO, milbemycin oxime; VAN, vancomycin; FDX, fidaxomicin.

### Cytotoxicity assessment against vero cells

Most of these hits are drugs that have been approved for human or veterinary use and their pharmacokinetics and toxicity profiles are well studied. For instance, dronedarone is approved for treatment of atrial fibrillation in humans with dosages up to 400 mg twice daily (83, 84). Broxyquinoline is also highly tolerable and its LD_50_ in mice is 7.4 g/kg when administered orally (85). Robenidine is generally regarded as a safe drug with no observed genotoxicity or mutagenicity (73). Although, milbemycin oxime and cholroquinaldol are approved drugs with low to no toxicity (61, 86). Also, ionomycin showed no toxicity on embryo development in mice (87). Additionally, we performed the *in vitro* cytotoxicity assessment for ET-770 and carbadox against monkey kidney epithelial (Vero) cells. The IC_50_ values of both drugs exceeded 128 µg/mL, indicating that Vero cells were able to tolerate them. This value indicates the high safety index for these metabolites, since it represents more than 1024 and 64 times the MIC_90_ of ET770 and carbadox, respectively, against strains of *C. difficile* (Fig. S1).

Additionally, most of these compounds have a relatively simple structure, such as carbadox, which can be further modified for optimization or to enhance specific anticlostridial activities, such as avoiding absorption from the gut, and reduce toxicity. The information we presented will provide an opportunity for medicinal chemists to further investigate these molecules and synthesize analogs with more potent activity and better pharmacokinetic characteristics, including reduced toxicity. This approach is standard in drug discovery

To summarize, screening a library of microbial metabolites led to the discovery of novel lead molecules, such as ionomycin (calcium salt), broxyquinoline, chlorquinaldol, and carbadox that possess potent *in vitro* anti-*C*. *difficile* activity and limited activity against some members of the gut normal microbiota. These metabolites warrant further investigation for the development of new anti-*C*. *difficile* therapeutics.

## SUPPLEMENTAL MATERIAL

Supplemental material msphere.00273-24-s0001.docx Tables S1 to S3 and Fig. S1.

ASM does not own the copyrights to Supplemental Material that may be linked to, or accessed through, an article. The authors have granted ASM a non-exclusive, world-wide license to publish the Supplemental Material files. Please contact the corresponding author directly for reuse.
