# Supplementary material for: Exploring novel microbial metabolites and drugs for inhibiting Clostridioides difficile
Source: mSphere. 2024 Jun 28;9(7):e00273-24. doi: 10.1128/msphere.00273-24 (PMC11288027; doi:10.1128/msphere.00273-24)
Supplement: Supplemental material — Tables S1 to S3 and Fig. S1. [file msphere.00273-24-s0001.docx]

# **Exploring Novel Microbial Metabolites and Drugs for Inhibiting *Clostridioides difficile***

Ahmed A. Abouelkhair^1,2,3^, and Mohamed N. Seleem^1,2#^

^1^ Department of Biomedical Sciences and Pathobiology, Virginia-Maryland College of Veterinary Medicine, Virginia Polytechnic Institute and State University; Blacksburg, VA, 24061, USA.

^2^ Center for One Health Research, Virginia Polytechnic Institute and State University, Blacksburg, VA 24061, USA.

^3^Department of Bacteriology, Mycology, and Immunology, Faculty of Veterinary Medicine, University of Sadat City, Sadat City, Menoufia, Egypt

#Address correspondence to Mohamed N. Seleem, naguieb@vt.edu

Department of Biomedical Sciences and Pathobiology

Virginia-Maryland College of Veterinary Medicine

Virginia Polytechnic Institute and State University

1410 Prices Fork Rd, Blacksburg, VA, 24061, USA

**Table S1: Full description of the source and the characters of *C. difficile* strains ( 20 Pathogenic strains):**

| No. | *C. difficile* Strain ID | Alternate designation | Source | Characters |
| --- | --- | --- | --- | --- |
| 1 | **CDI -1** | *Clostridium difficile* Isolate1067 | Obtained from the CDC.  Isolated from Homosapien (Unknown) in 2016, USA (Ribotype -027). | It is positive for *tcdA, tcdB, cdtA, and cdtB* |
| 2 | **CDI -6** | *Clostridium difficile* Isolate1072 | Obtained from the CDC.  Isolated from Homosapien (Unknown) in 2016, USA (Ribotype -027). | It is positive for *tcdA, tcdB, cdtA, and cdtB* |
| 3 | **CDI -10** | *Clostridium difficile* Isolate1076 | Obtained from the CDC.  Isolated from Homosapien (Unknown) in 2016, USA (Ribotype -027). | It is positive for *tcdA, tcdB, cdtA, and cdtB* |
| 4 | **CDI -12** | *Clostridium difficile* Isolate1078 | Obtained from the CDC.  Isolated from Homosapien (Unknown) in 2016, USA | It is positive for *tcdA, tcdB,* but negative with *cdtA, cdtB* |
| 5 | **CDI -13** | *Clostridium difficile* Isolate1079 | Obtained from the CDC.  Isolated from Homosapien (Unknown) in 2016, USA | It is positive for *tcdA, tcdB,* but negative with *cdtA, cdtB* |
| 6 | **CDI -15** | *Clostridium difficile* Isolate1081 | Obtained from the CDC.  Isolated from Homosapien (Unknown) in 2016, USA | It is positive for *tcdA, tcdB,* but negative with *cdtA, cdtB* |
| 7 | **CDI -22** | *Clostridium difficile* Isolate1088 | Obtained from the CDC.  Isolated from Homosapien (Unknown) in 2016, USA | It is positive for *tcdA, tcdB,* but negative with *cdtA, cdtB* |
| 8 | **CDI -23** | *Clostridium difficile* Isolate1089 | Obtained from the CDC.  Isolated from Homosapien (Unknown) in 2016, USA | It is positive for *tcdA, tcdB,* but negative with *cdtA, cdtB* |
| 9 | **CDI -24** | *Clostridium difficile* Isolate1090 | Obtained from the CDC.  Isolated from Homosapien (Unknown) in 2016, USA | It is positive for *tcdA, tcdB,* but negative with *cdtA, cdtB* |
| 10 | **CDI -26** | *Clostridium difficile* Isolate1092 | Obtained from the CDC.  Isolated from Homosapien (Unknown) in 2016, USA (Ribotype -027). | It is positive for *tcdA, tcdB, cdtA, and cdtB* |
| 11 | **CDI -28** | *Clostridium difficile* Isolate1094 | Obtained from the CDC.  Isolated from Homosapien (Unknown) in 2016, USA | It is positive for *tcdA, tcdB,* *cdtA,* and *cdtB* |
| 12 | **CDI -29** | *Clostridium difficile* Isolate1095 | Obtained from the CDC.  Isolated from Homosapien (Unknown) in 2016, USA (Ribotype -027). | It is positive for *tcdA, tcdB, cdtA, and cdtB* |
| 13 | **CDI-30** | *Clostridium difficile* Isolate1096 | Obtained from the CDC.  Isolated from Homosapien (Unknown) in 2016, USA | It is positive for *tcdA, tcdB,* but negative with *cdtA, cdtB* |
| 14 | **NR- 49302** | *Clostridium difficile* Isolate 20111075 | Obtained from the BEI Resources.  Isolated from the stool of an elderly male patient with a healthcare-associated (HA) *C. difficile* infection in Minnesota, USA, in 2010. | Containing *tcdA*, *tcdB* and *tcdC* of the PaLoc operon. This isolate is reported to be negative for the *C. difficile* binary toxin (CDT). |
| 15 | **NR- 49304** | *Clostridium difficile* Isolate 20120956 | Obtained from the BEI Resources.  Isolated from the stool of an older male patient with a healthcare-associated (HA) *C. difficile*infection in southern USA in 2011. | Containing *tcdA, tcdB*and *tcdC* of the PaLoc operon. This isolate is reported to be negative for the *C. difficile* binary toxin (CDT). |
| 15 | **NR- 49308** | *Clostridium difficile* Isolate 20120166 | Obtained from the BEI Resources.  Isolated from the stool of an elderly female patient with a community-associated (CA) *C. difficile* infection in Tennessee, USA, in 2011. | Containing *tcdA*, *tcdB* and *tcdC* of the PaLoc operon. This isolate is reported to be negative for the *C. difficile* binary toxin (CDT). |
| 17 | **NR- 49319** | *Clostridium difficile* Isolate 20110992 | Obtained from the BEI Resources.  Isolated from the stool of an elderly male patient with a community-associated (CA) *C. difficile* infection in midwestern USA in 2011. | Containing *tcdA*, *tcdB* and *tcdC* of the PaLoc operon. This isolate is reported to be negative for the *C. difficile* binary toxin (CDT). |
| 18 | **ATCC BAA-1870** | *Clostridium difficile* Isolate4118 | Obtained from the ATCC.  Clinical isolate (Ribotype - 027) | It is positive for *tcdA, tcdB,* and *cdtB* |
| 19 | **ATCC 630** | *Clostridium difficile* IsolateBAA-1382 | Obtained from the ATCC.  Isolated from Switzerland | It is positive for *tcdA, tcdB,* but negative with *cdtB* |
| 20 | **ATCC 43255** | *Clostridium difficile* Isolate VPI 10463 | Obtained from the ATCC.  Isolated from abdominal wound | It is positive for *tcdA, tcdB,* but negative with *cdtB* |

**CDC**; The Centers for Disease Control and Prevention.

**BEI Resources**; The Biodefense and Emerging Infections Research Resources Repository.

**ATCC**; The American Type Culture Collection.

**Table S2: Full description of the source and the characters of gut microbiota (15 strains):**

| No. | Bacterial Strain ID | Alternate designation | Source |
| --- | --- | --- | --- |
| 1 | ***Bacteroides fragilis***  **HM-709** | *Bacteroides fragilis* CL07T00C01 | Obtained from the BEI Resources.  Isolated from healthy adult feces in Massachusetts, USA. |
| 2 | ***Bacteroides fragilis***  **HM-718** | *Bacteroides dorei* CL03T12C01 | Obtained from the BEI Resources.  Isolated from healthy adult human feces in Boston, Massachusetts, USA. |
| 3 | ***Bacteroides dorei***  **HM-719** | *Bacteroides dorei* CL02T12C06 | Obtained from the BEI Resources.  Isolated from healthy adult human feces in Boston, Massachusetts, USA. |
| 4 | ***Bifidobacterium breve***  **ATCC 15700** | *Bifidobacterium breve*  S1 (Variant a) | Obtained from the ATCC.  Isolated from intestine |
| 5 | ***Bifidobacterium***  ***angulatum***  **HM- 1189** | *Bifidobacterium angulatum*  F16_22 | Obtained from the BEI Resources.  Isolated from human stool in Guelph, Ontario, Canada. |
| 6 | ***Bifidobacterium adolescentis* HM-633** | *Bifidobacterium adolescentis* L2-32 | Obtained from the BEI Resources.  Isolated in 1996 from the fecal sample of a healthy two-year-old infant in Aberdeen, Scotland, United Kingdom. |
| 7 | ***Bifidobacterium longum* HM-845** | *Bifidobacterium longum subsp. longum* 44B | Obtained from the BEI Resources.  Isolated from a one-year-old human patient. |
| 8 | ***Bifidobacterium longum* HM-846** | *Bifidobacterium longum subsp. longum* 1-6B | Obtained from the BEI Resources.  Isolated in 2006 from feces of a six-year-old healthy human child in Russia. |
| 91 | ***Bifidobacterium longum***  **HM- 847** | *Bifidobacterium longum subsp. Longum* 35B | Obtained from the BEI Resources.  Isolated from a one-year-old human patient. |
| 10 | ***Bifidobacterium longum***  **HM- 848** | *Bifidobacterium longum subsp. Longum* 2-2B | Obtained from the BEI Resources.  Isolated from a six-year-old human patient. |
| 11 | ***Enterococcus faecalis***  **HM 202** | *Enterococcus faecalis*  TX1322 | Obtained from the BEI Resources.  Isolated in 1994 from the feces of a community volunteer in Texas, USA. |
| 12 | ***Enterococcus faecium***  **HM 970** | *Enterococcus faecium*  ERV165 | Obtained from the BEI Resources.  Isolated from human feces collected in Colombia, in 2008. |
| 13 | ***Lacticaseibacillus paracasei* ATCC 334** | **--** | Obtained from the ATCC  Isolated from dairy products; emmental cheese |
| 14 | ***Levilactobacillus brevis***  **ATCC-14869** | *Lactobacillus brevis* Bb14 | Obtained from the ATCC.  Isolated from feces |
| 15 | ***Lactobacillus rhamnoosus***  **ATCC-53103** |  | Obtained from the ATCC.  Isolated from feces |

**BEI Resources**; The Biodefense and Emerging Infections Research Resources Repository.

**ATCC**; The American Type Culture Collection.

**Table S3: Detailed explanation of all 63 compounds that were shown to have strong hits against *C. difficile* during library screening.**

| No. | Metabolite ID | Chemical structure | Indication |
| --- | --- | --- | --- |
| 1 | **Tunicamycin** | 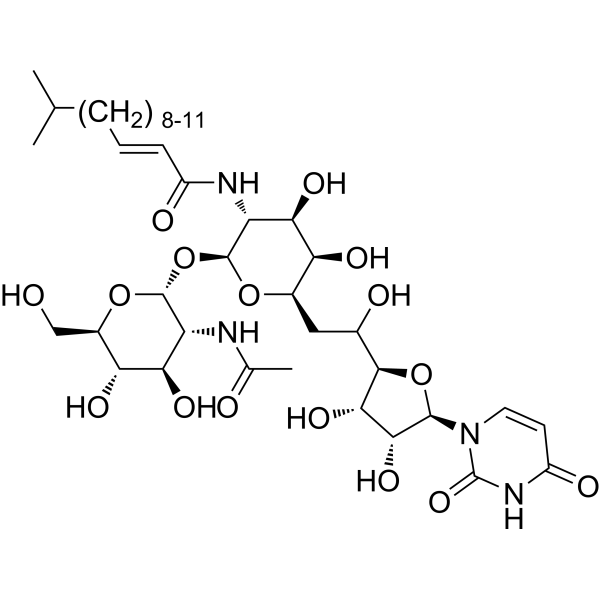 | Antibiotic; Bacterial; Fungal; Influenza Virus |
| 2 | **Ecteinascidin 770** | 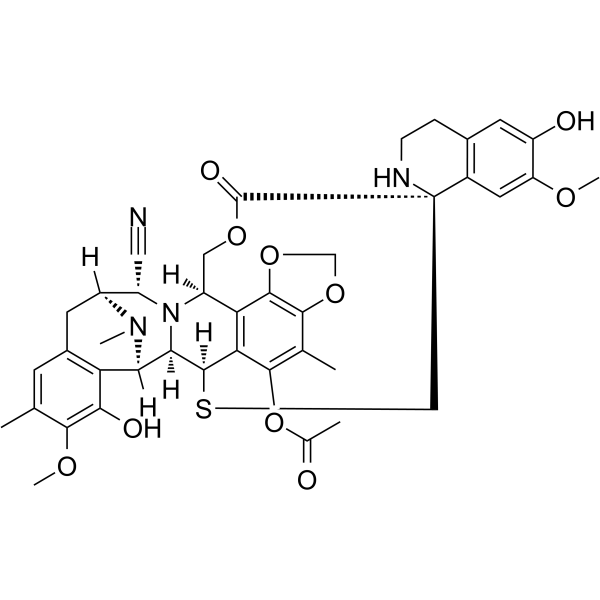 | Apoptosis |
| 3 | **Nanchangmycin** | 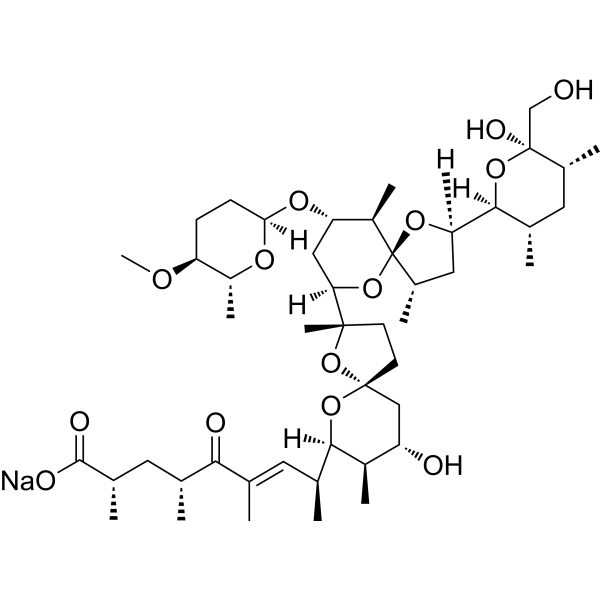 | Antibacterial |
| 4 | **Avermectin B1** | 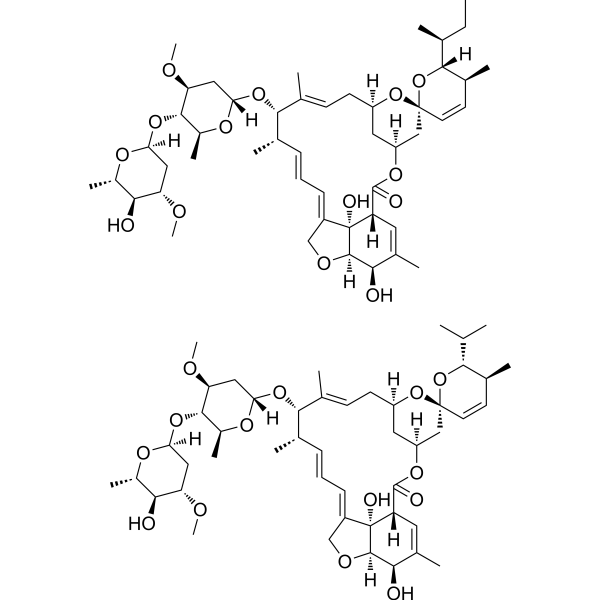 | Anthelmintic |
| 5 | **GW 501516** | 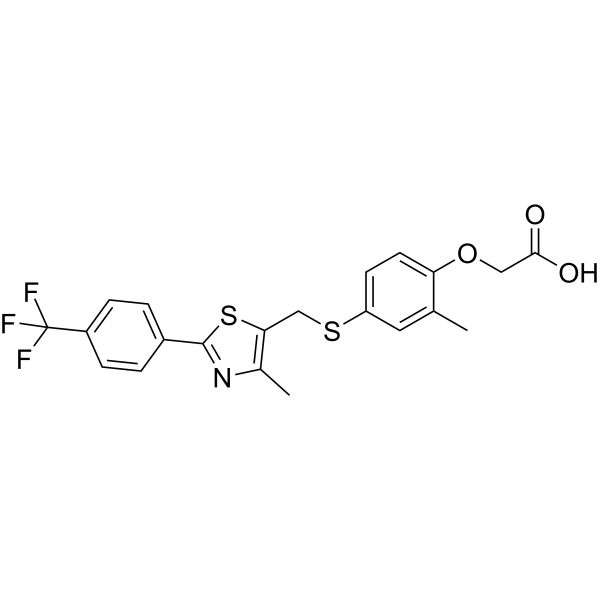 | Metabolic modulator |
| 6 | **Rifamycin S** | 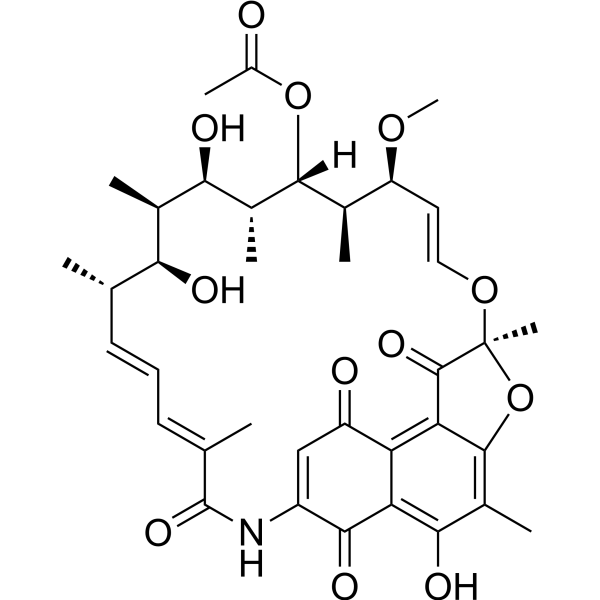 | Antibiotic; Bacterial; Reactive Oxygen Species |
| 7 | **Ionomycin (calcium)** | 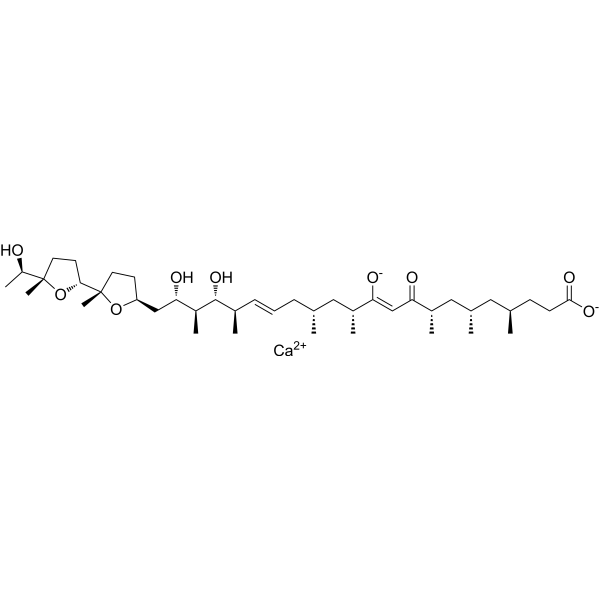 | Calcium ionophore |
| 8 | **Ivermectin** | 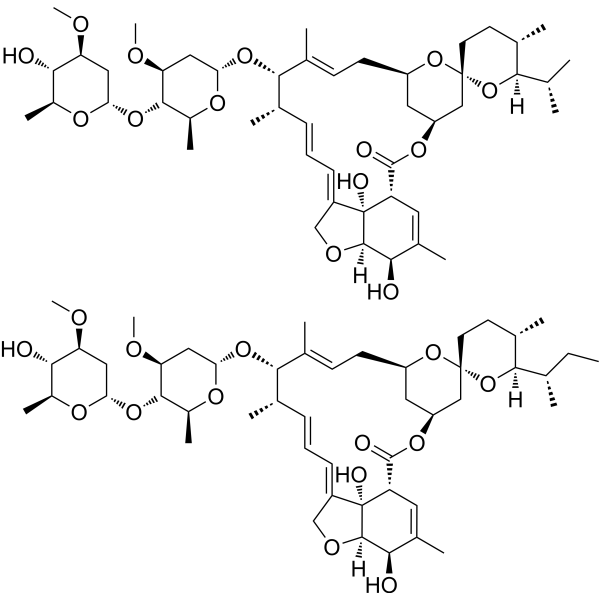 | Anthelmintic |
| 9 | **Dianemycin** | 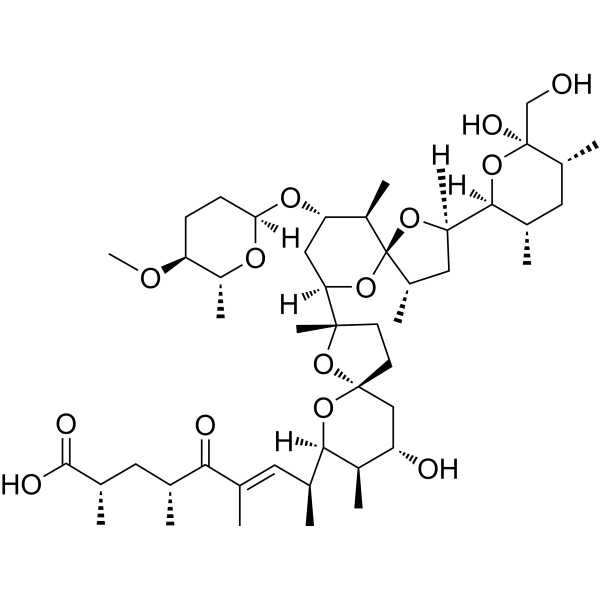 | Antibacterial |
| 10 | **Salinomycin** | 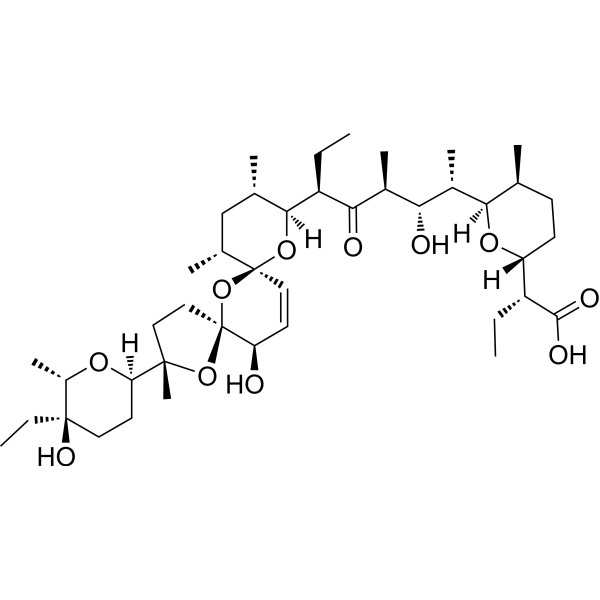 | Antibiotic; Apoptosis; Autophagy; Bacterial; Mitophagy; Wnt; β-catenin |
| 11 | **Reutericyclin** | 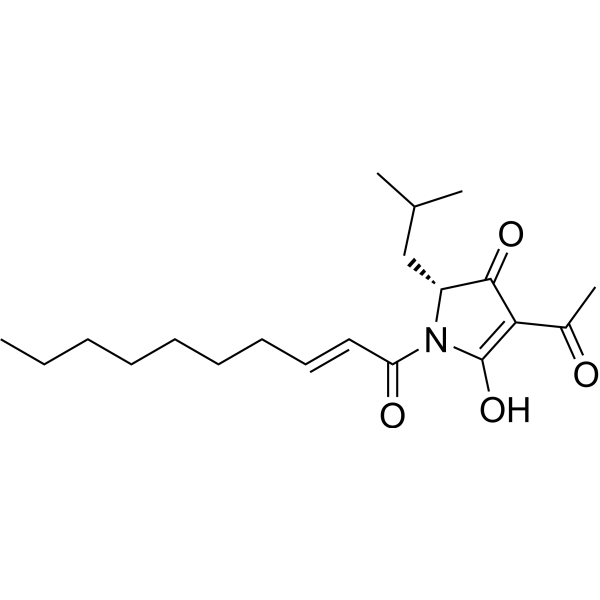 | Antibiotic; Bacterial |
| 12 | **Dronedarone** | 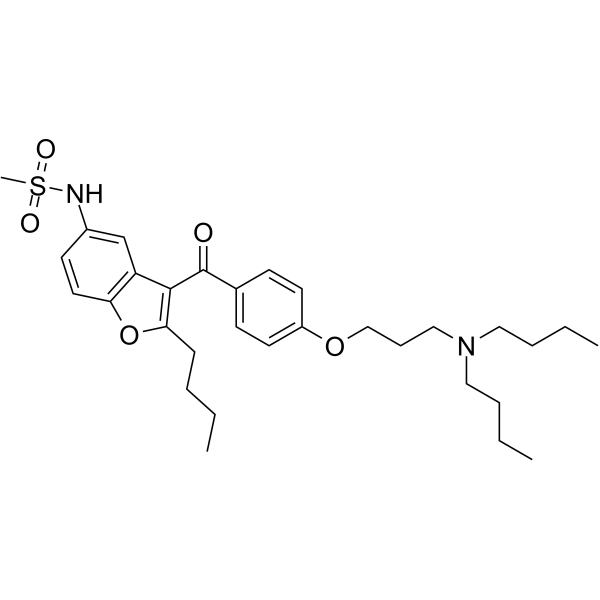 | Adrenergic Receptor; Autophagy; Calcium Channel; Cytochrome P450; mAChR; Sodium Channel |
| 13 | **Daptomycin** | 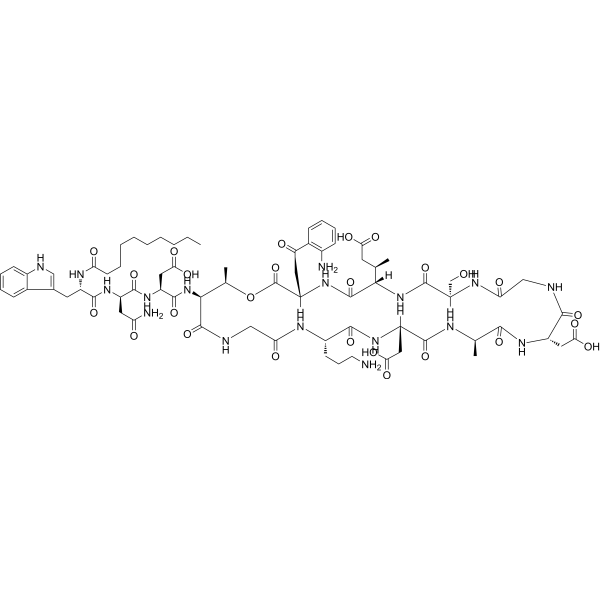 | Antibacterial |
| 14 | **Vancomycin (hydrochloride)** | 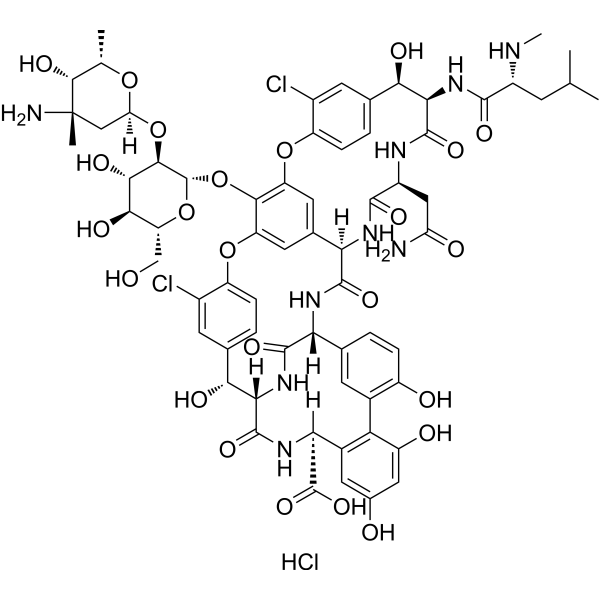 | Antibacterial |
| 15 | **Metronidazole** | 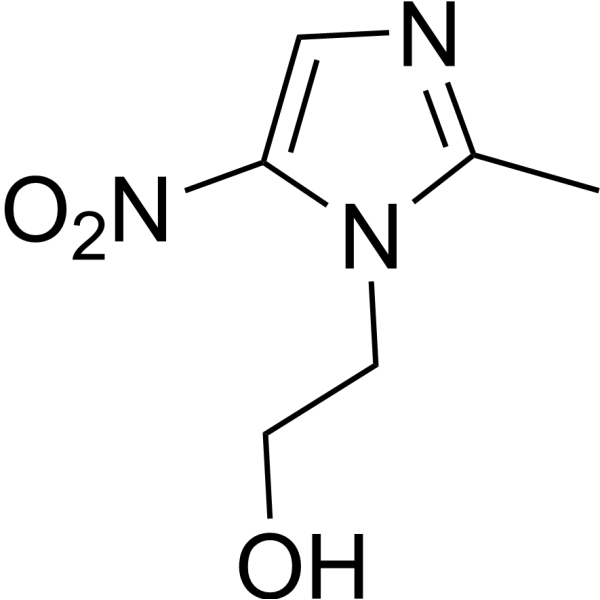 | Antibacterial, and antiparasitic |
| 16 | **Chloroxine** | 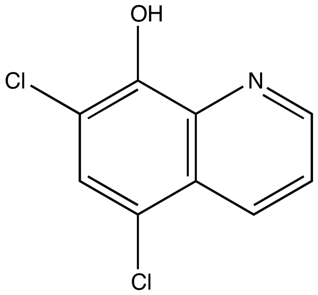 | Chelating agent, anti-seborrheic |
| 17 | **Ochromycinone** | 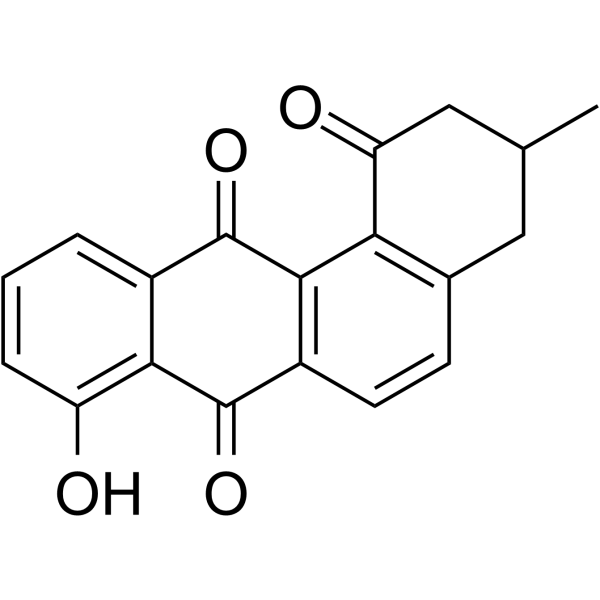 | (Rac)-STA-21 Bacterial; STAT |
| 18 | **Fidaxomicin** | 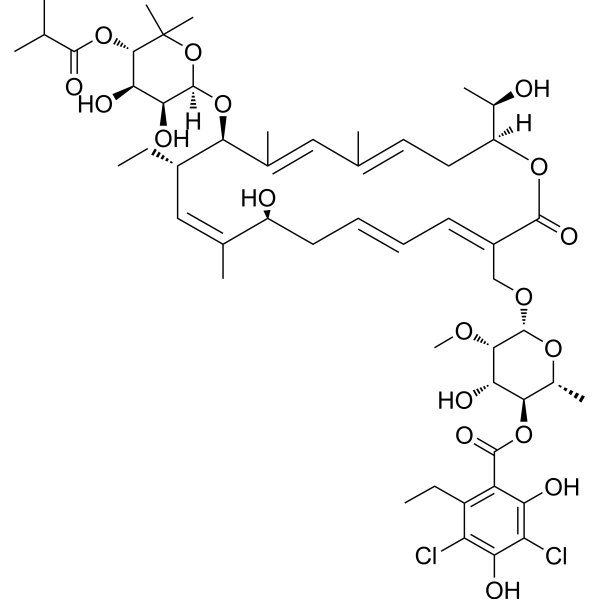 | Antibacterial |
| 19 | **Tioconazole** | 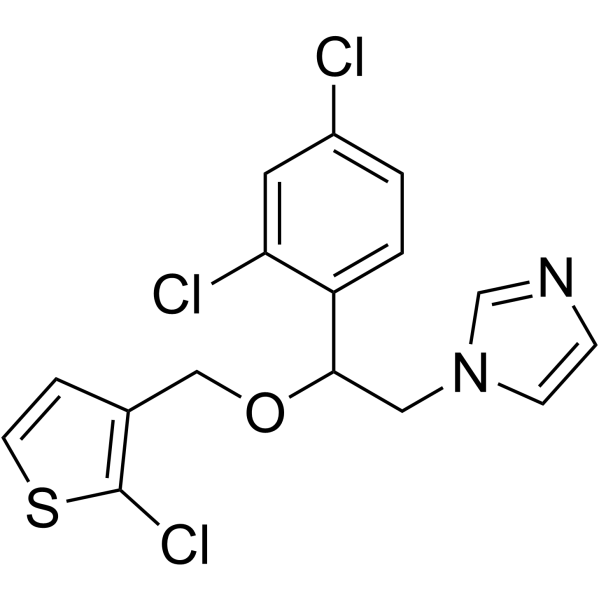 | Antifungal |
| 20 | **Demeclocycline (hydrochloride)** | 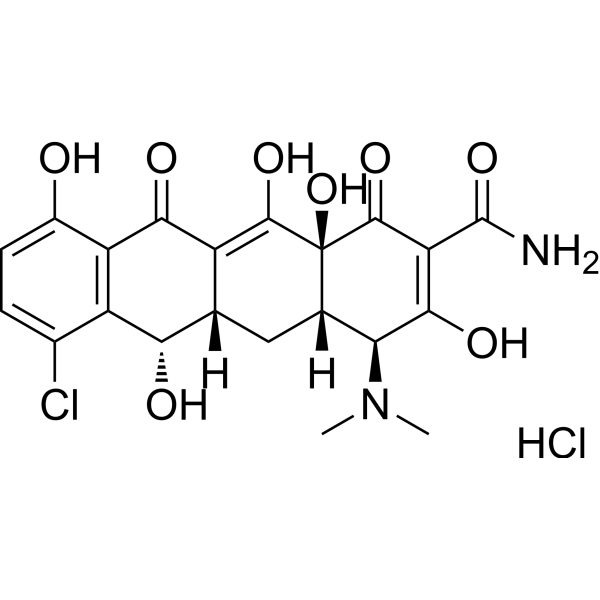 | Antibacterial |
| 21 | **Nitrofurazone** | 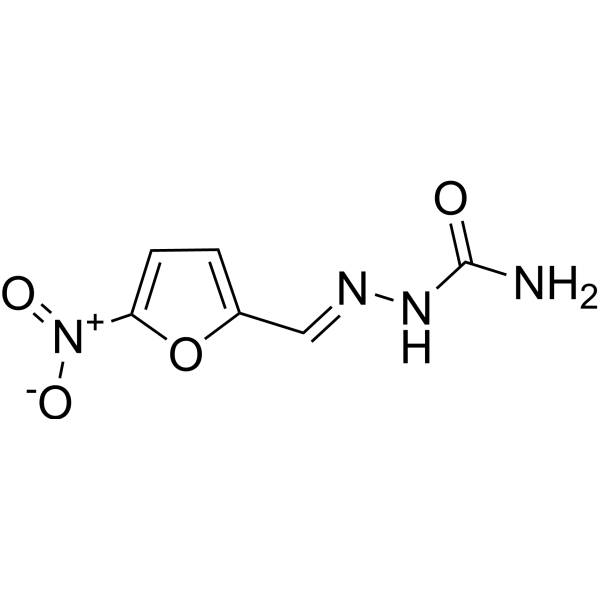 | Antibacterial |
| 22 | **Lithocholic acid** | 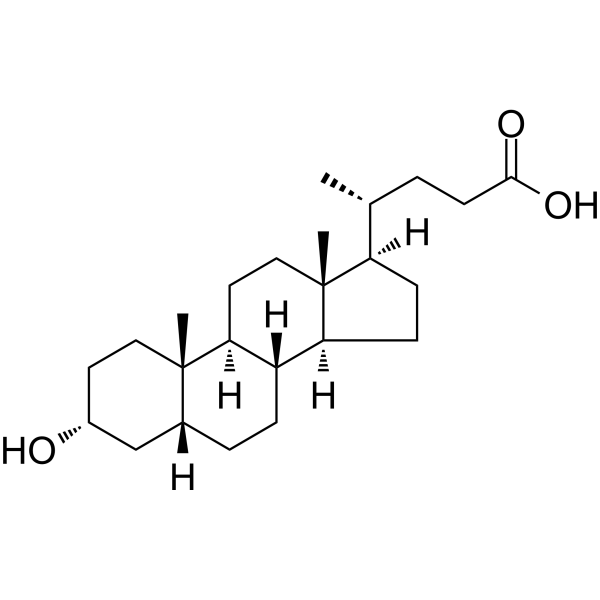 | Apoptosis; Autophagy; Endogenous Metabolite |
| 23 | **Tinidazole** | 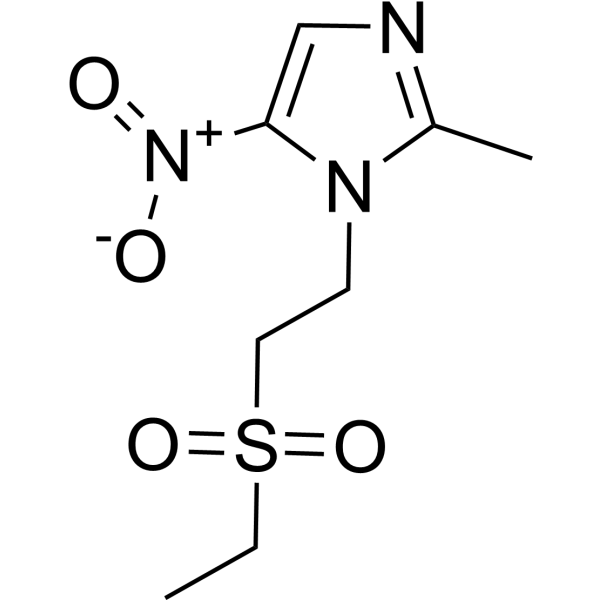 | Antiprotozoal |
| 24 | **Valnemulin (hydrochloride)** | 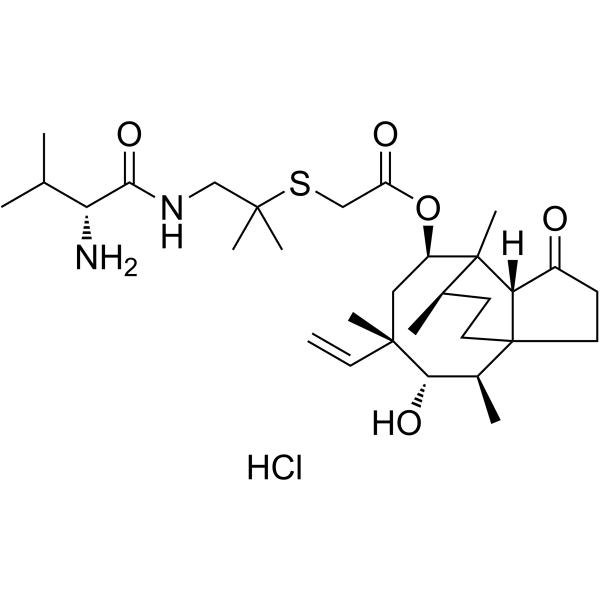 | Antibacterial |
| 25 | **Oxytetracycline (hydrochloride)** | 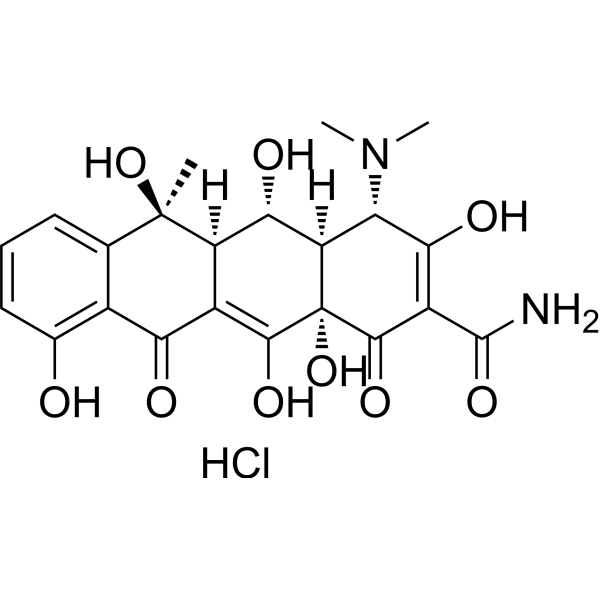 | Antibacterial; Endogenous Metabolite; HSV |
| 26 | **Vitamin B12** | 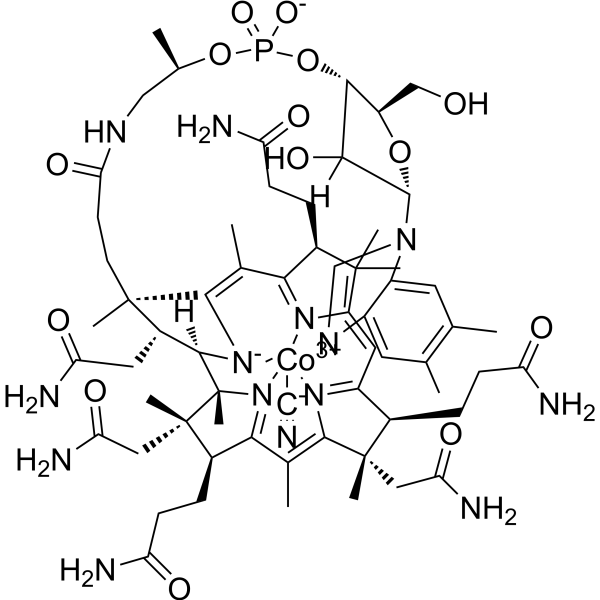 | Endogenous Metabolite |
| 27 | **Oxytetracycline** | 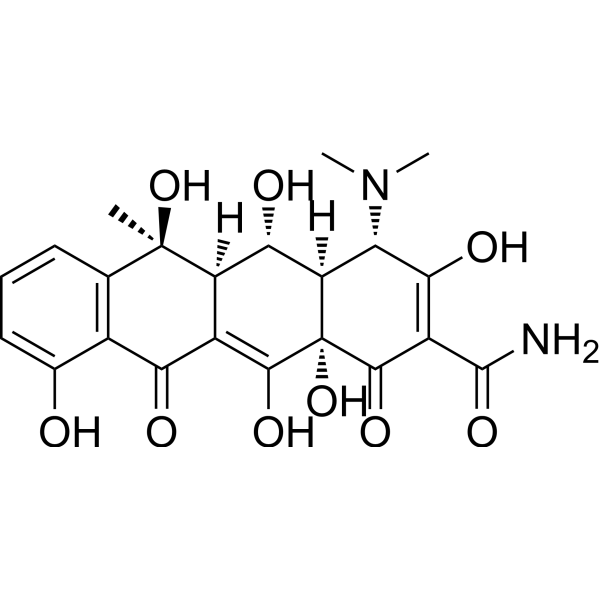 | Antibacterial; Endogenous Metabolite; HSV |
| 28 | **Ornidazole** | 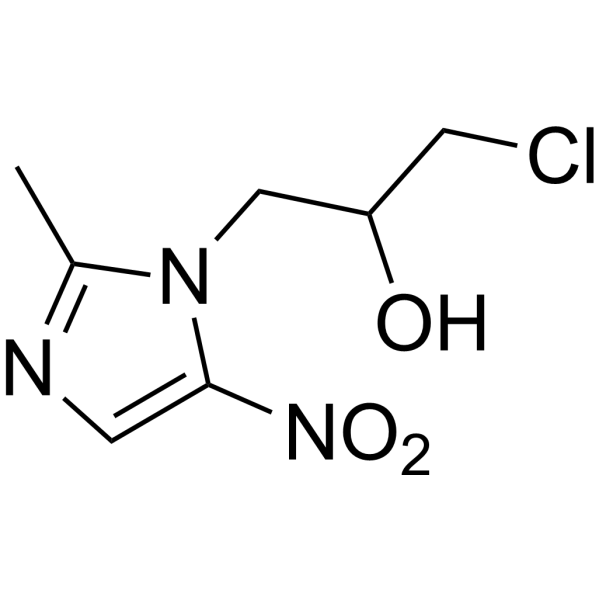 | Antibacterial, and antiparasitic |
| 29 | **Nithiamide** | 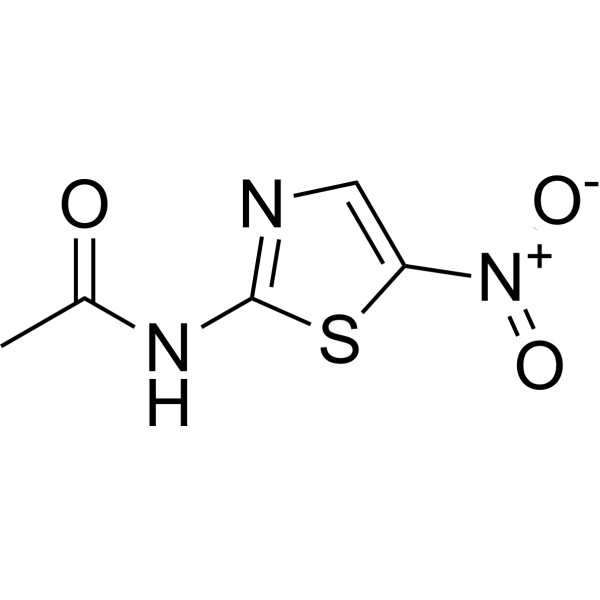 | Antibacterial |
| 30 | **Thiostrepton** | 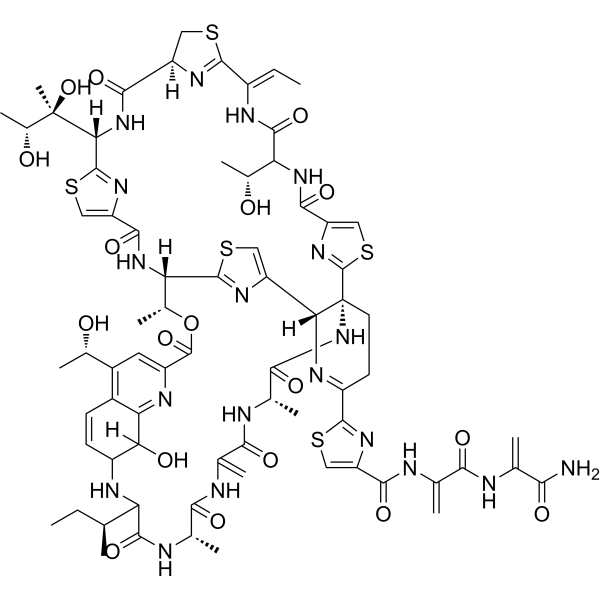 | Antibacterial |
| 31 | **Lasalocid (sodium)** | 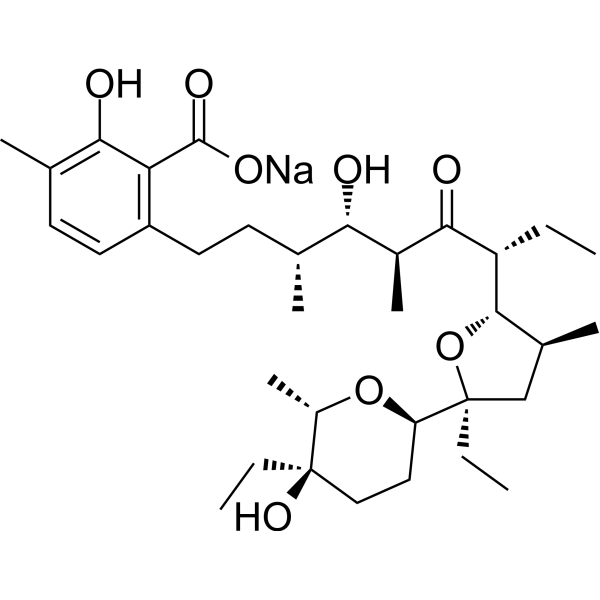 | Antibacterial |
| 32 | **Furaltadone (hydrochloride)** | 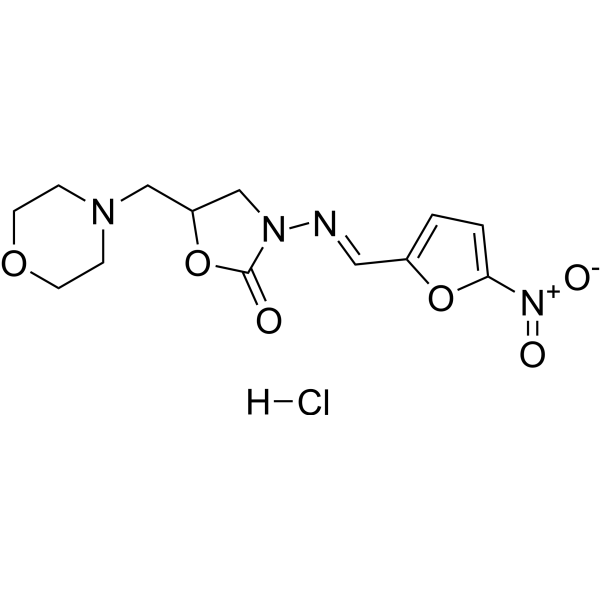 | Antibacterial |
| 33 | **Tricaprilin** | 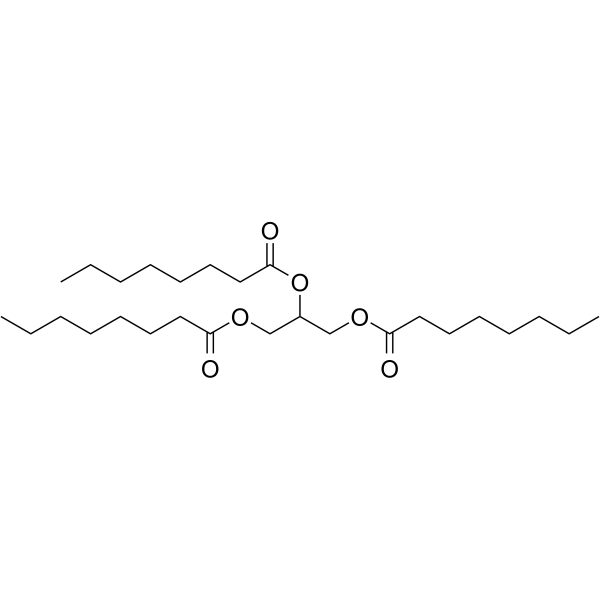 | Endogenous Metabolite |
| 34 | **Milbemycin oxime** | 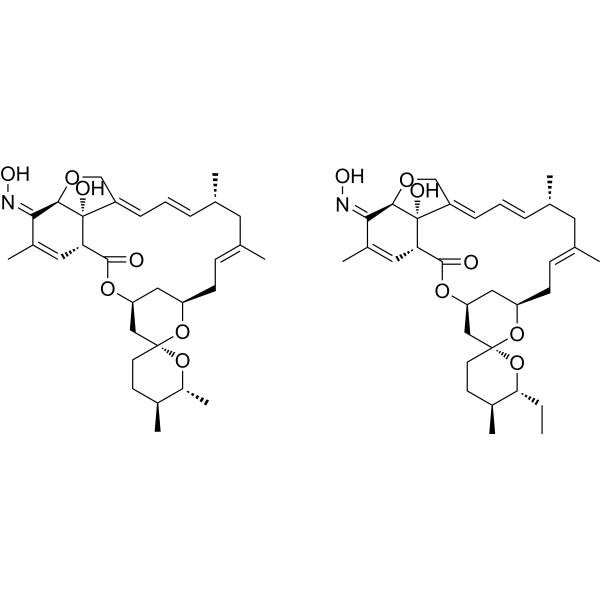 | Anthelmintic |
| 35 | **Sitafloxacin (hydrate)** | 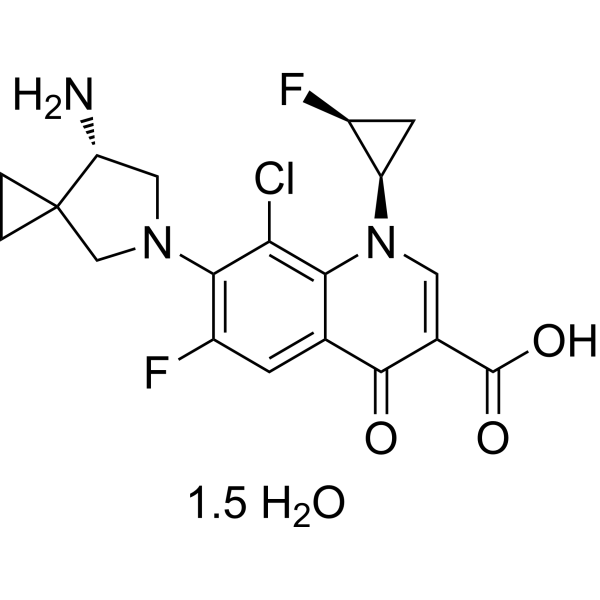 | Antibacterial |
| 36 | **Tylosin (tartrate)** | 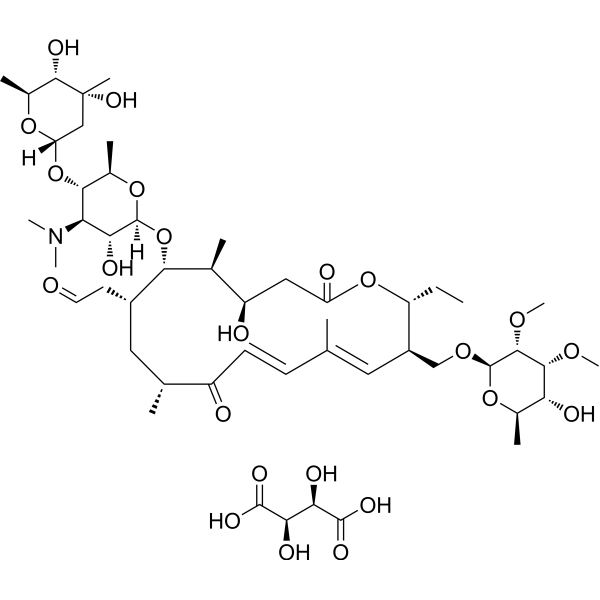 | Antibacterial |
| 37 | **Broxyquinoline** | 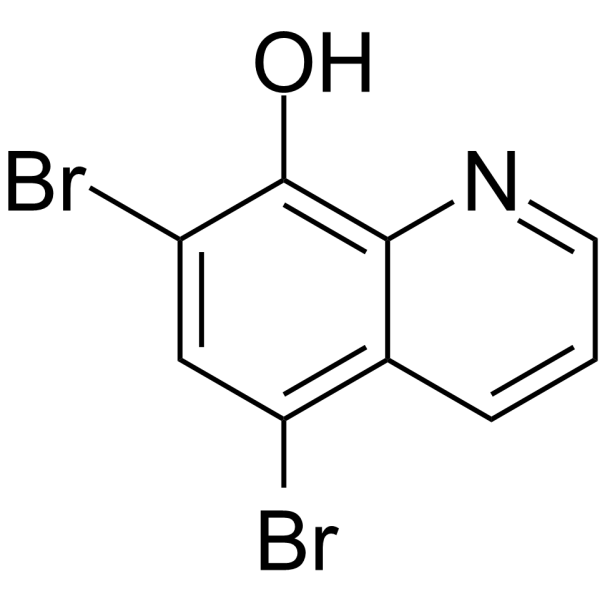 | Antiprotozoal |
| 38 | **L-Cycloserine** | 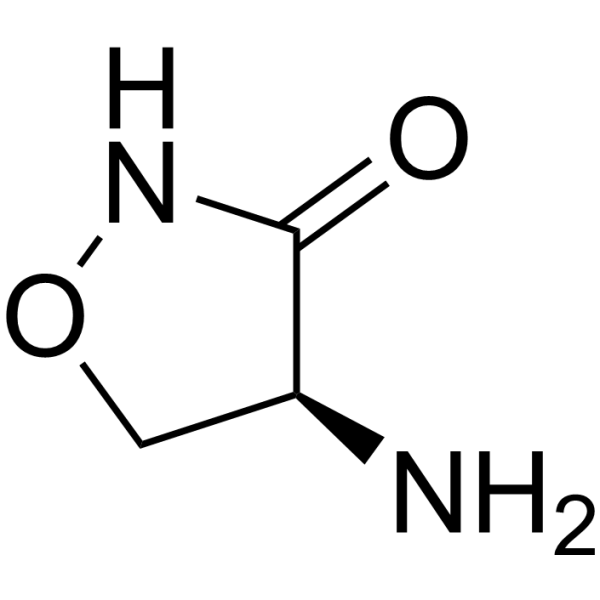 | GABA Receptor; HIV |
| 39 | **Furazolidone** | 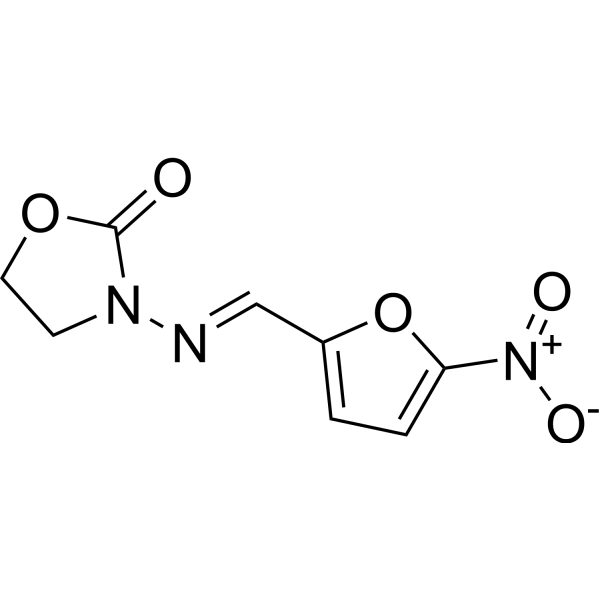 | Antibiotic; Apoptosis; Bacterial |
| 40 | **Chlorquinaldol** | 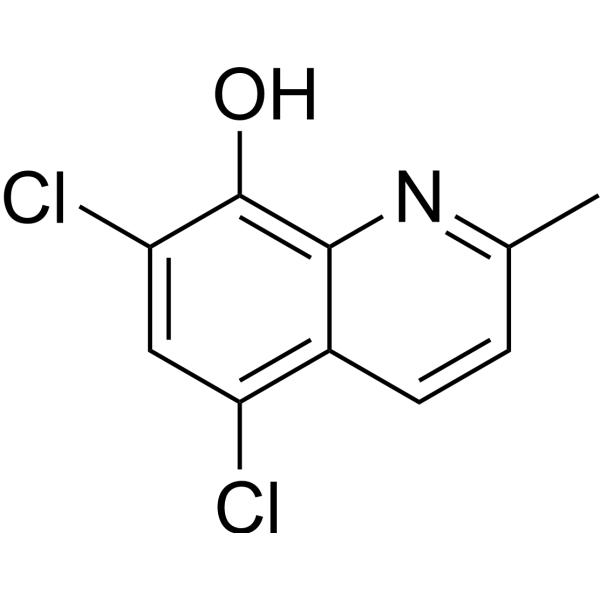 | Topical antiseptic and for vaginal infections |
| 41 | **Tetracycline (hydrochloride)** | 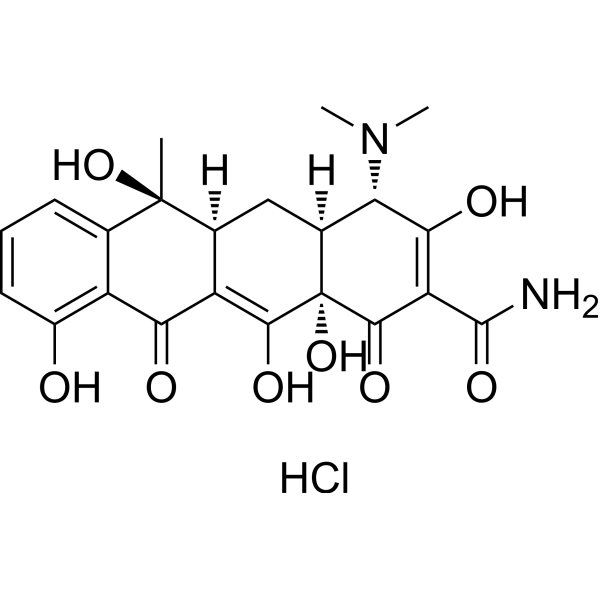 | Antibacterial |
| 42 | **Aklomide** | 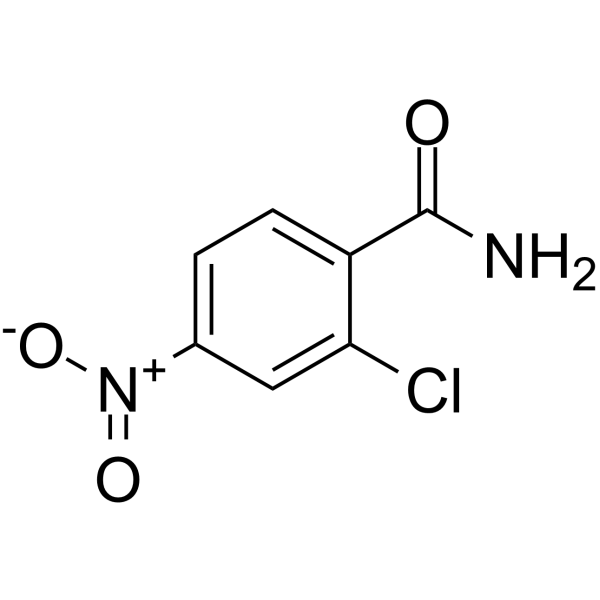 | Parasite |
| 43 | **Tylosin** | 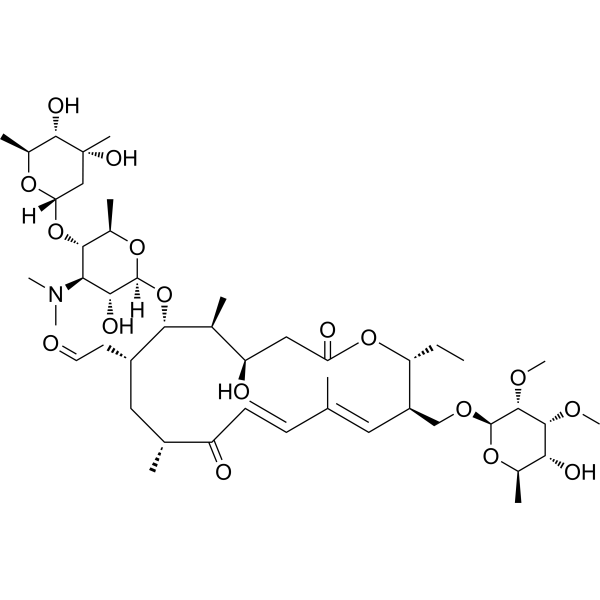 | Antibacterial |
| 44 | **Miconazole (nitrate)** | 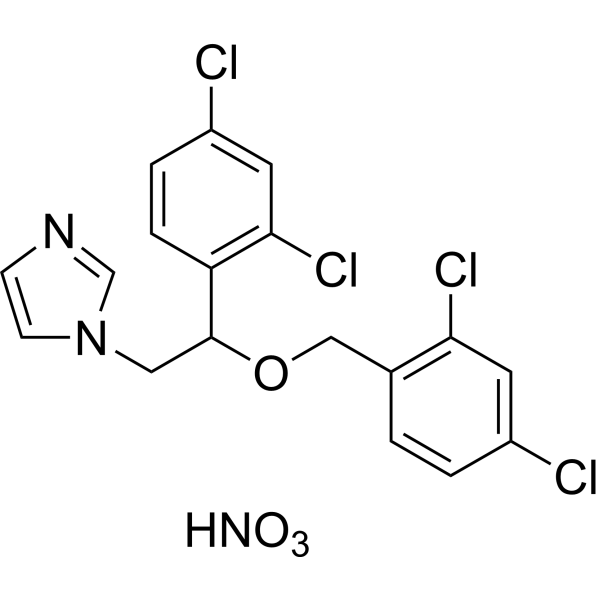 | Antifungal |
| 45 | **Carbadox** | 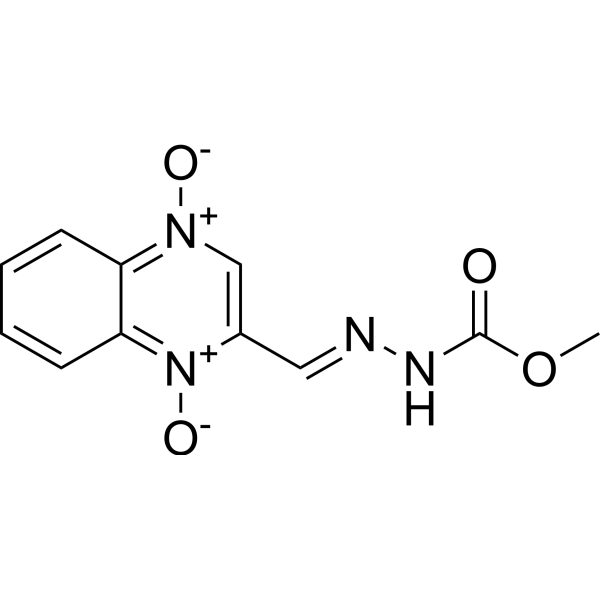 | Feed additive (Growth promoter)  Endogenous Metabolite |
| 46 | **Puromycin (dihydrochloride)** | 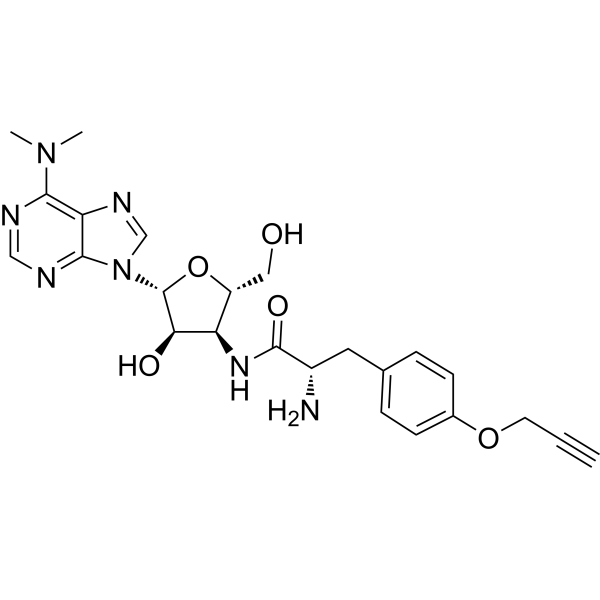 | Antibacterial |
| 47 | **Hygromycin B** | 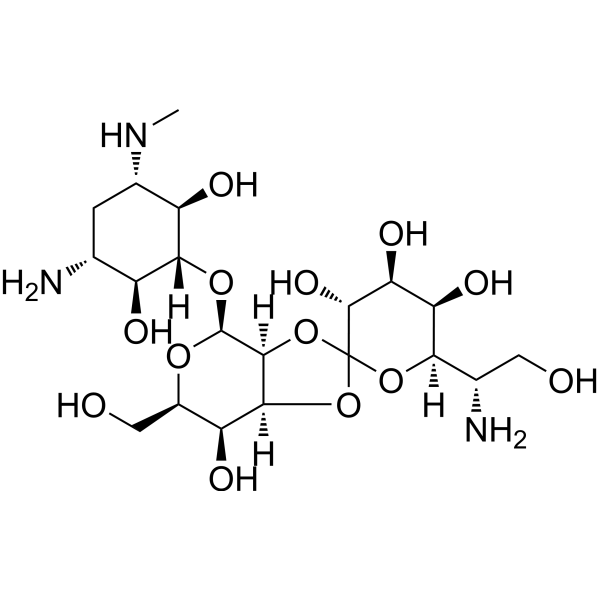 | Antibiotic; Bacterial; Fungal |
| 48 | **Triclosan** | 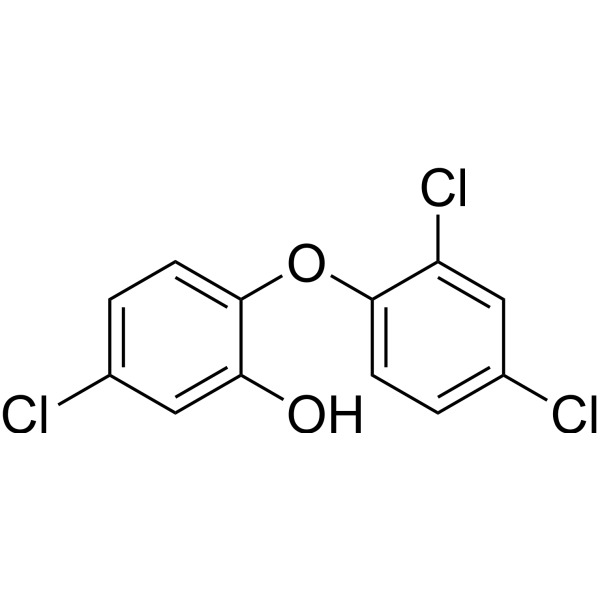 | Antibiotic; Autophagy; Bacterial; Fungal |
| 49 | **Fusidic acid (sodium salt)** | 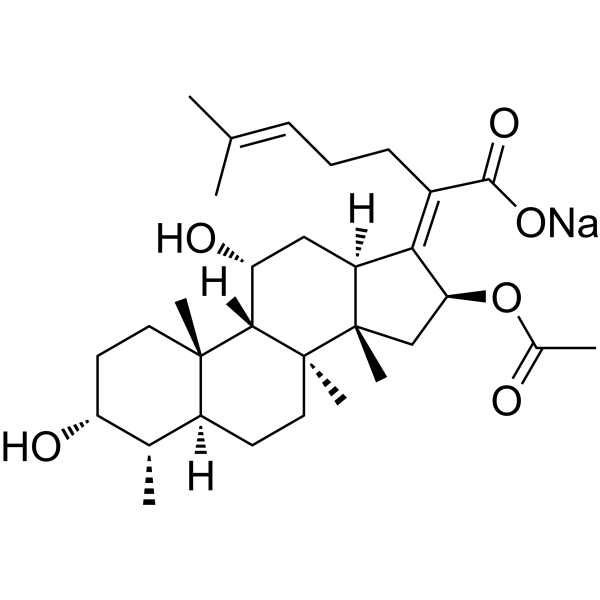 | Antibacterial |
| 50 | **Dihydroergotoxine (mesylate)** | 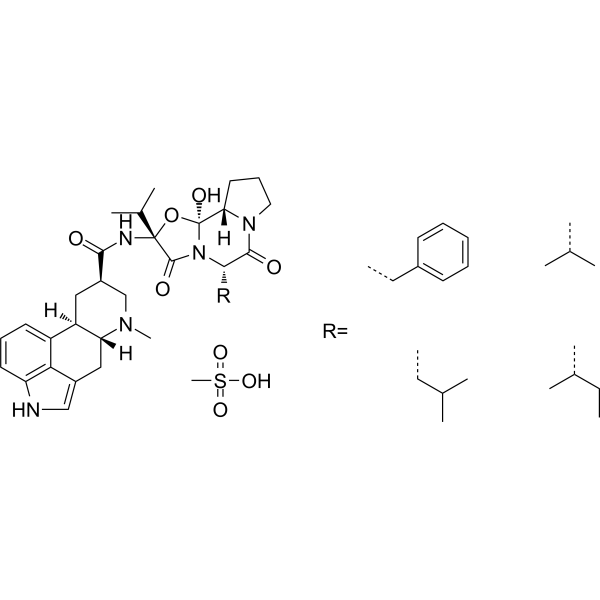 | GABA Receptor |
| 51 | **Ginsenoside C-K** | 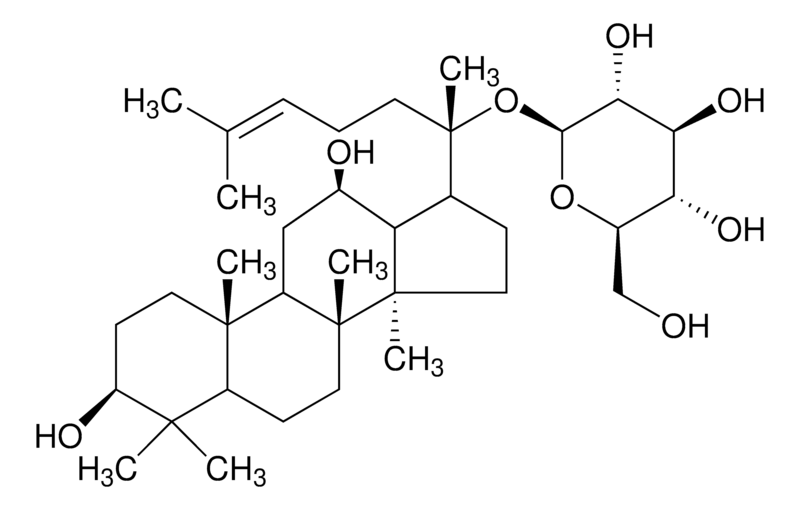 | COX; Cytochrome P450; NO Synthase  Anti-inflammatory and anti-neoplastic |
| 52 | **Azomycin** | 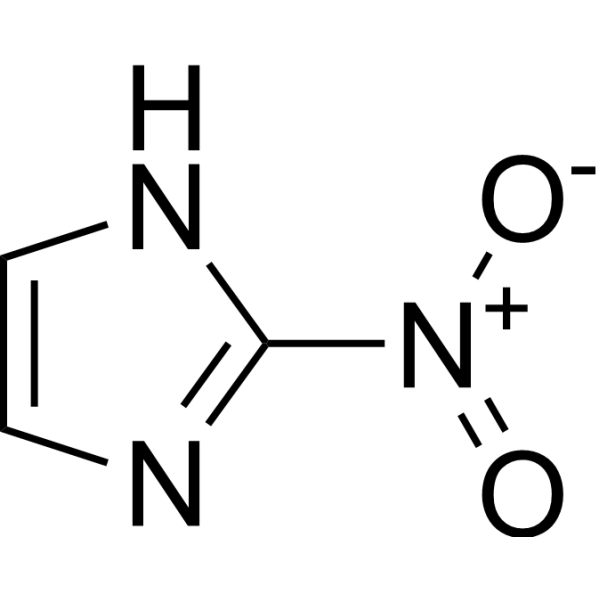 | Antibacterial |
| 53 | **Doxycycline (hyclate)** | 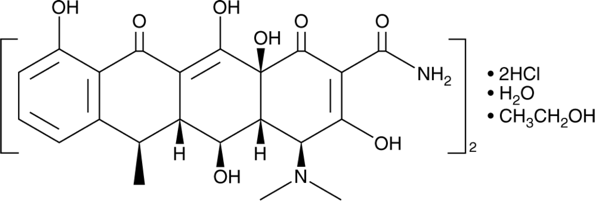 | Antibacterial |
| 54 | **Robenidine hydrochloride** | 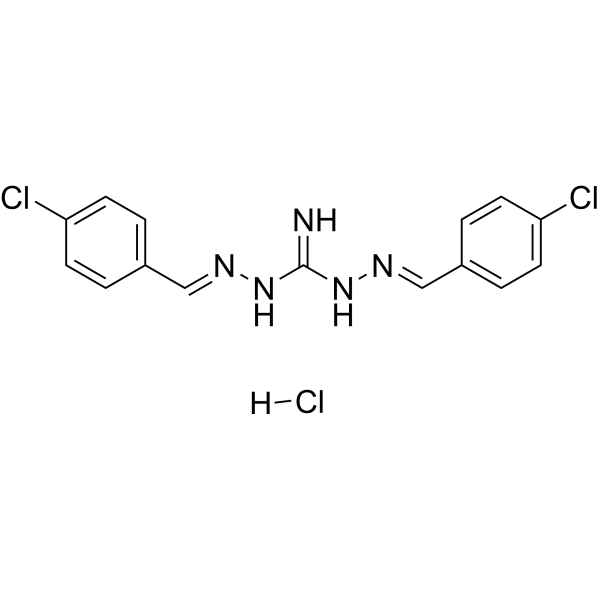 | Coccidiostat drug |
| 55 | **Pleuromutilin** | 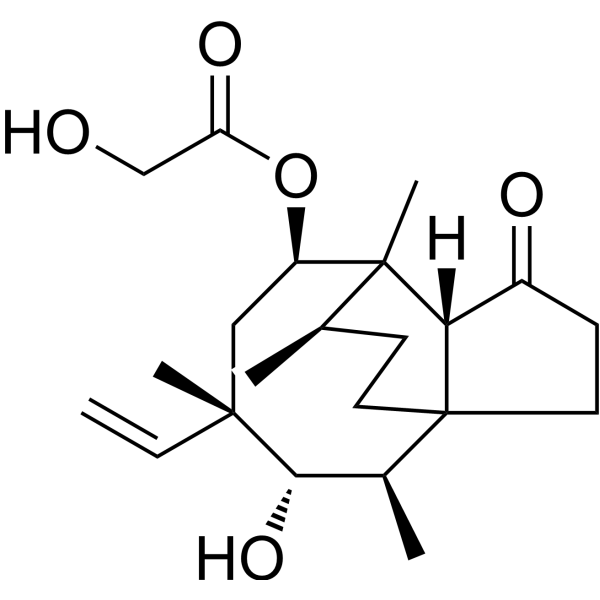 | Antibacterial |
| 56 | **Gramicidin** | 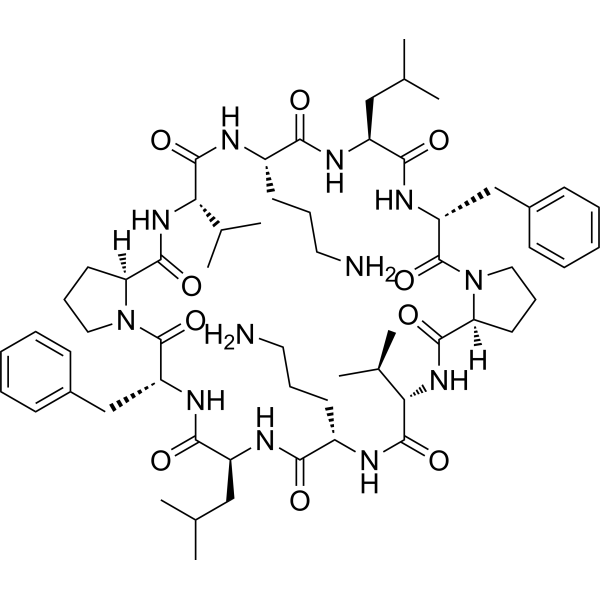 | Antibacterial |
| 57 | **Valinomycin** | 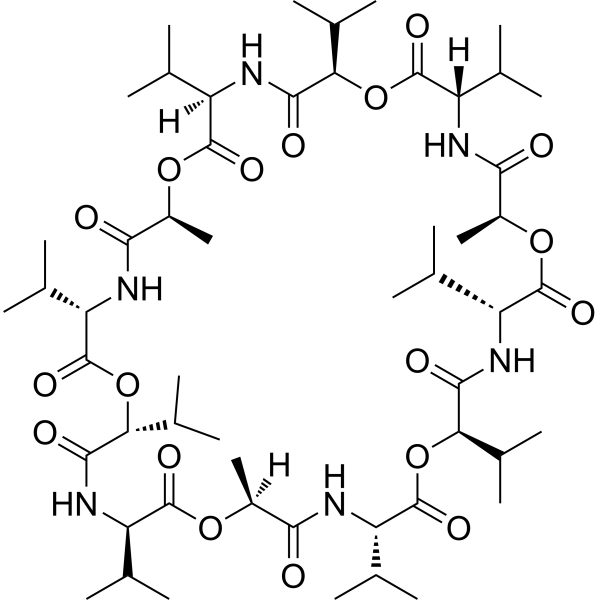 | Antibiotic; Apoptosis; Autophagy; Bacterial |
| 58 | **Sultamicillin (tosylate)** | 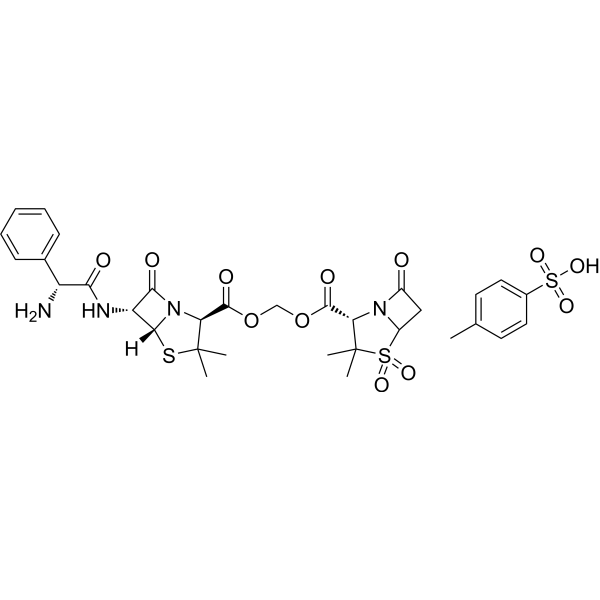 | Antibacterial |
| 59 | **Clofoctol** | 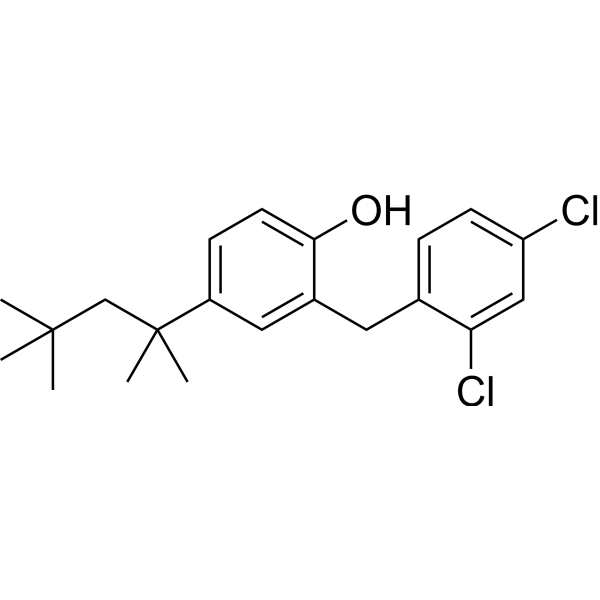 | Antibacterial |
| 60 | **Chlortetracycline (hydrochloride)** | 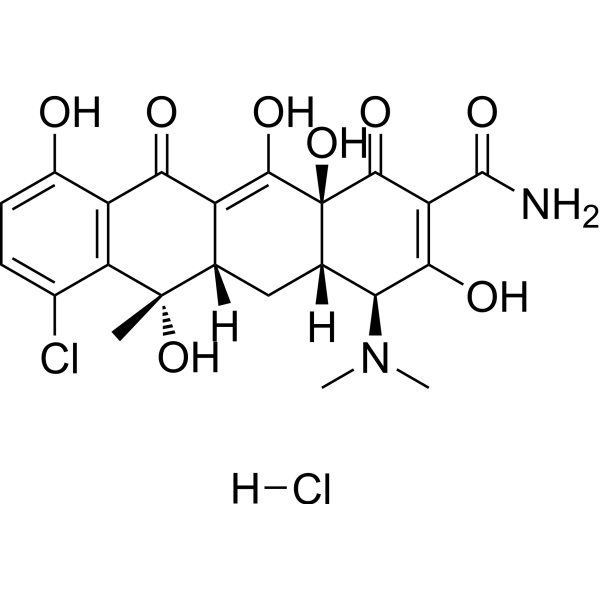 | Antibacterial |
| 61 | **Octenidine (dihydrochloride)** | 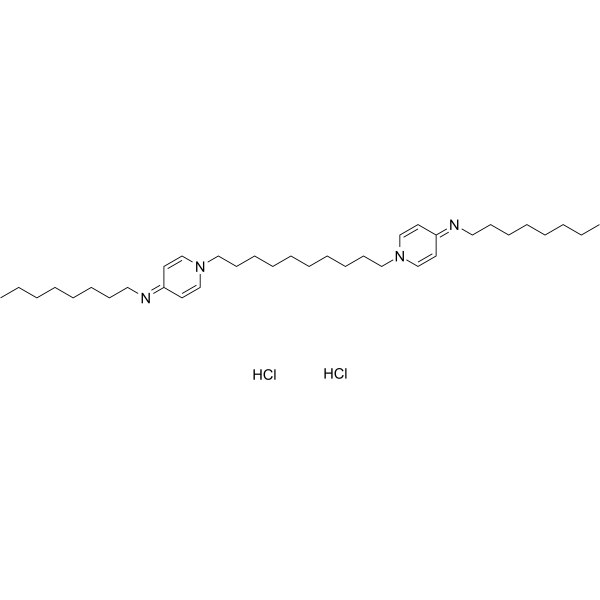 | Antiseptic |
| 62 | **Nigericin (sodium salt)** | 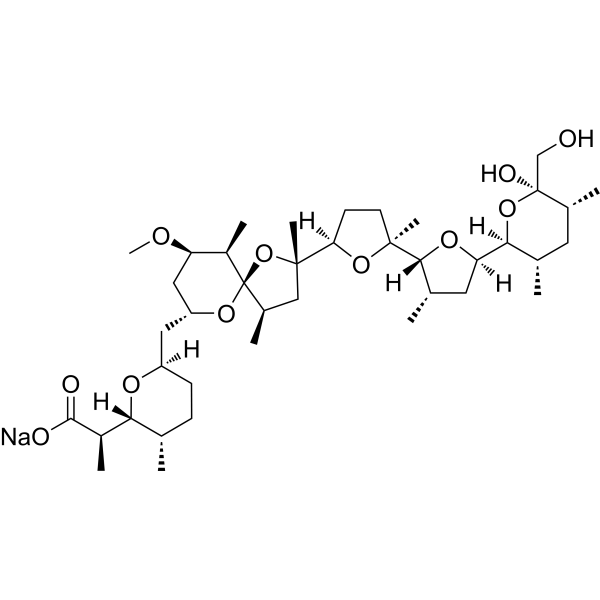 | Antibacterial; NOD-like Receptor (NLR); Potassium Channel |
| 63 | **Monensin sodium salt** | 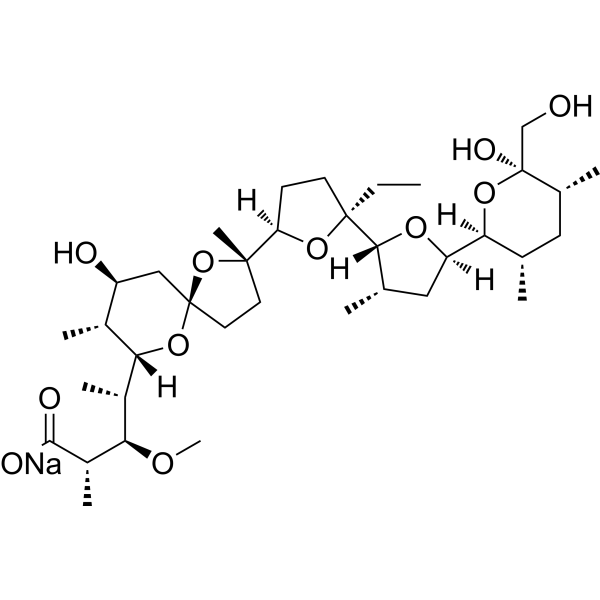 | Antibacterial; Sodium Channel |


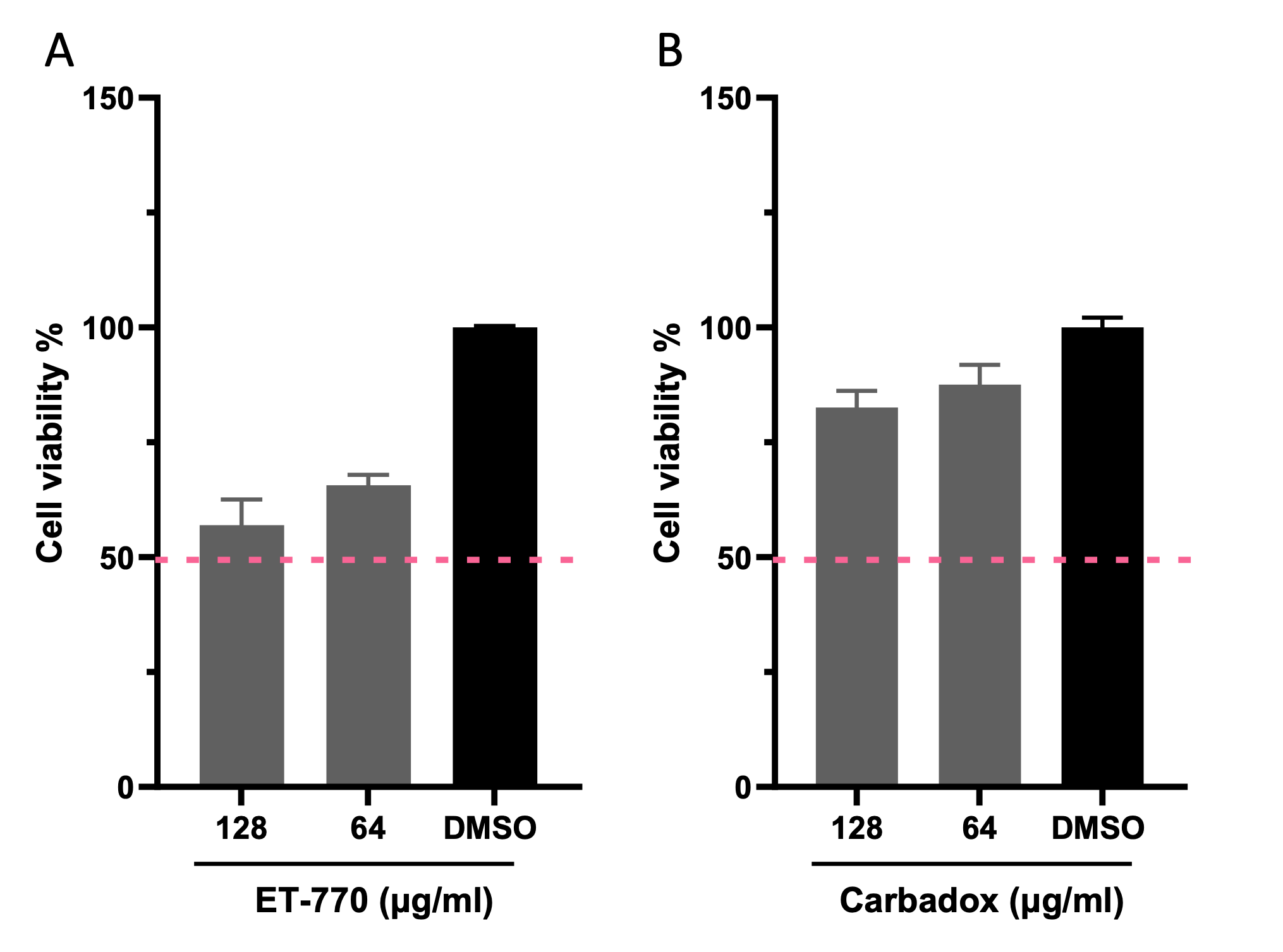


**Figure S1. Cell cytotoxicity activity against the Vero cell line was tested.** When utilizing the MTS3-(4,5-dimethylthiazol-2-yl)-5-(3-carboxymethoxyphenyl)-2-(4-sulfophenyl)-2H tetrazolium) test to examine the cytotoxicity of **A)** ET-770 and **B)** carbadox beginning at 128 $\mu$g/ml, the percentage of live Vero cells is calculated as a ratio of average absorbance relative to DMSO.
